# Supplementary material for: Synthesis and Antiparasitic Activity of New Trithiolato-Bridged Dinuclear Ruthenium(II)-arene-carbohydrate Conjugates
Source: Molecules. 2023 Jan 16;28(2):902. doi: 10.3390/molecules28020902 (PMC9865825; doi:10.3390/molecules28020902)
Supplement: Supplementary file 1 [file molecules-28-00902-s001.zip › molecules-2094676-supplementary.pdf]

Supplementary Materials

Synthesis and Antiparasitic Activity of New Trithiolato-Bridged Dinuclear Ruthenium(II)-Arene - Carbohydrate Conjugates

Isabelle Holzer [1], Oksana Desiatkina [1], Nicoleta Anghel [2], Serena K. Johns [1,3], Ghalia Boubaker [2], Andrew Hemphill [2], Julien Furrer [1] and Emilia Păunescu [1]

1 Department of Chemistry, Biochemistry and Pharmaceutical Sciences, University of Bern, Freiestrasse 3, 3012 Bern, Switzerland.
2 Institute of Parasitology Vetsuisse Faculty, University of Bern, Länggass-Strasse 122, 3012 Bern, Switzerland;
3 School of Chemistry, Cardiff University, Park Place, Cardiff CF103AT, UK

Table of contents

	Page
**Experimental chemistry**	S2
**1. General**	S2
**2. Synthesis of the trithiolato-bridged dinuclear ruthenium(II)-arene intermediates 2-9**	S2
**3. Synthesis of the azide and alkyne functionalized carbohydrate intermediates 10-18**	S3
**4. Synthesis of the carbohydrate functionalized trithiolato-bridged dinuclear ruthenium(II)-arene complexes 19-26**	S8
**Stability in DMSO-*d*₆**	S18
**Figure S1.** ^{1}H NMR Spectra of **6**, **7**, **20**, **22**, **25** and **26** recorded in DMSO-d_6 at 25°C; (A) recorded 5 min after sample preparation, and (B) sample after > 30 days storage at 0-5°C in the dark.	S19
**References**	S20

Experimental chemistry

1. General

Chemicals were purchased from Aldrich, Alfa Aesar, Acros Organics, ABCR, and TCI Chemicals and used without further purification. Reactions were performed under inert atmosphere (N_2) using Schlenk techniques with dry solvents (Acros Organics) preserved over molecular sieves. ^{1}H (400.13 MHz) and ^{13}C (100.62 MHz) NMR spectra were recorded on a Bruker Avance II 400 spectrometer at 298 K. The chemical shifts are reported in parts per million (ppm) and referenced to residual solvent peaks [1] ($CDCl_3$, ^{1}H δ 7.26, $^{13}C\{^{1}H\}$ δ 77.16 ppm; DMSO-d_6, ^{1}H δ 2.50, $^{13}C\{^{1}H\}$ δ 39.52 ppm), and coupling constants (J) are reported in hertz (Hz). High resolution electrospray ionization mass spectra (ESI-MS) were carried out by the Mass Spectrometry and Protein Analyses Services at DCBP and were obtained on a LTQ Orbitrap XL ESI (Thermo) operated in positive ion mode. Thermal elemental analyses were carried out by the Mass Spectrometry and Protein Analyses Services at DCBP and were obtained on a Flash 2000 Organic Elemental Analyzer (Thermo Scientific). Reactions were monitored by TLC using Merck TLC silica gel coated aluminium sheets 60 F254 and visualized with UV at 254 nm, and KMnO4 stain. Compounds were purified by column flash chromatography on silica gel using the elution systems indicated.

Abbreviations

$BF_3{\cdot}Et_2O$ – Boron trifluoride diethyl etherate
DMAP – 4-(Dimethylamino)-pyridine
DMF – Dimethylformamide
EDCI – *N*-(3-Dimethylaminopropyl)-*N′*-ethylcarbodiimide hydrochloride
EtOAc – Ethyl acetate
Hex – *n*-Hexane
TBAF – Tetrabutylammonium fluoride
THF – Tetrahydrofuran
For the description of the NMR spectra: *Ar* – arene, *Tr* – triazole.

2. Synthesis of the trithiolato-bridged dinuclear ruthenium(II)-arene intermediates 2-9

The dithiolato intermediate **1** ([(η^6-*p*-$MeC_6H_4Pr^i$)$_2Ru_2$(μ_2-$SCH_2C_6H_4$-*p*-Bu^t)$_2Cl_2$]) was prepared and purified by adapting a previously published protocol [2,3].

Compounds **2** ([(η^6-*p*-$MeC_6H_4Pr^i$)$_2Ru_2$(μ_2-$SCH_2C_6H_4$-*p*-Bu^t)$_2$(μ_2-SC_6H_4-*p*-OH)]Cl), **3** ([(η^6-*p*-$MeC_6H_4Pr^i$)$_2Ru_2$(μ_2-$SCH_2C_6H_4$-*p*-Bu^t)$_2$(μ_2-SC_6H_4-*p*-NH_2)]Cl), **4** ([(η^6-*p*-$MeC_6H_4Pr^i$)$_2Ru_2$(μ_2-$SCH_2C_6H_4$-*p*-Bu^t)$_2$(μ_2-SC_6H_4-*p*-CH_2CO_2H)]Cl), and **5** ([(η^6-*p*-$MeC_6H_4Pr^i$)$_2Ru_2$(μ_2-$SCH_2C_6H_4$-*p*-Bu^t)$_2$(μ_2-SC_6H_4-*o*-CH_2OH)]Cl) were synthetised and purified according to previously reported protocols [2-4].

The synthesis of compounds **7** ([(η^6-*p*-$MeC_6H_4Pr^i$)$_2Ru_2$(μ_2-$SCH_2C_6H_4$-*p*-Bu^t)$_2$(μ_2-SC_6H_4-*p*-NH-R)]Cl, R = (C=O)-$(CH_2)_3$-C≡CH), **8** ([(η^6-*p*-$MeC_6H_4Pr^i$)$_2Ru_2$(μ_2-$SCH_2C_6H_4$-*p*-Bu^t)$_2$(μ_2-SC_6H_4-*p*-CH_2-

(C=O)-NH-R)]Cl, R = CH$_2$-C≡CH) and **9** ([(η^6-*p*-MeC$_6$H$_4$Pr^i)$_2$Ru$_2$(μ_2-SCH$_2$C$_6$H$_4$-*p*-Bu^t)$_2$(μ_2-SC$_6$H$_4$-*o*-CH$_2$-N$_3$)]Cl), was described in detail in precedent reports [5,6].

Synthesis of [(η^6-*p*-MeC$_6$H$_4$Pr^i)$_2$Ru$_2$(μ_2-SCH$_2$C$_6$H$_4$-*p*-Bu^t)$_2$(μ_2-SC$_6$H$_4$-*p*-O-R)]Cl (R = (C=O)-(CH$_2$)$_3$-C≡CH) (6)

To a solution of 5-hexynoic acid (0.095 g, 0.847 mmol, 1.2 equiv.) in dry CH$_2$Cl$_2$ (40 mL) at r.t. under inert atmosphere (N$_2$) were successively added **2** (0.700 g, 0.706 mmol, 1 equiv.), EDCI (0.271 g, 1.412 mmol, 2 equiv.) and DMAP (0.043 g, 0.353 mmol, 0.5 equiv.). The reaction mixture was stirred at r.t. for 24 h and then concentrated to dryness under reduced pressure. Purification by column chromatography using CH$_2$Cl$_2$/CH$_3$OH 10:1 (v/v) as eluent afforded **6** as an orange solid (0.360 g, 0.332 mmol, yield 47%).

^{1}H-NMR (CDCl$_3$) δ_H, ppm: 7.79 (2H, d, 2xS-(*Ar*)C-C$\underline{H}$-CH-C-O, $^3J_{H,H}$ = 8.5 Hz), 7.39-7.50 (8H, m, 4xCH$_2$-(*Ar*)C-C$\underline{H}$-CH-C-C(CH$_3$)$_3$, 4xS-CH$_2$-(*Ar*)C-CH-C$\underline{H}$-C-C(CH$_3$)$_3$), 7.06 (2H, d, 2xS-(*Ar*)C-CH-C$\underline{H}$-C-O, $^3J_{H,H}$ = 8.5 Hz), 5.14 (2H, d, 2xCH$_3$-(*Ar*)C-C$\underline{H}$-CH-C, $^3J_{H,H}$ = 5.7 Hz), 5.02 (2H, d, 2xCH$_3$-(*Ar*)C-CH-C$\underline{H}$-C, $^3J_{H,H}$ = 5.8 Hz), 4.93 (2H, d, 2xCH$_3$-(*Ar*)C-CH-C$\underline{H}$-C, $^3J_{H,H}$ = 5.7 Hz), 4.63 (2H, d, 2xCH$_3$-(*Ar*)C-C$\underline{H}$-CH-C, $^3J_{H,H}$ = 5.8 Hz), 3.61 (2H, s, 2xS-C$\underline{H_2}$-(*Ar*)C-CH-CH-C-C(CH$_3$)$_3$), 3.44 (2H, s, 2xS-C$\underline{H_2}$-(*Ar*)C-CH-CH-C-C(CH$_3$)$_3$), 2.74 (2H, t, (C=O)-C$\underline{H_2}$-(CH$_2$)$_2$-C≡CH, $^3J_{H,H}$ = 7.4 Hz), 2.36 (2H, td, (C=O)-(CH$_2$)$_2$-C$\underline{H_2}$-C≡CH, $^3J_{H,H}$ = 6.8 Hz, $^4J_{H,H}$ = 2.6 Hz), 2.03 (1H, t, (C=O)-(CH$_2$)$_3$-C≡C$\underline{H}$, $^4J_{H,H}$ = 2.6 Hz), 1.97 (2H, qvint, (C=O)-CH$_2$-C$\underline{H_2}$-CH$_2$-C≡CH, $^3J_{H,H}$ = 7.2 Hz), 1.83-1-93 (2H, m, 2x(*Ar*)C-CH-CH-C-C$\underline{H}$(CH$_3$)$_2$), 1.77 (6H, s, 2xC$\underline{H_3}$-(*Ar*)C-CH-CH-C), 1.36 (9H, s, S-CH$_2$-(*Ar*)C-CH-CH-C-C(C$\underline{H_3}$)$_3$), 1.33 (9H, s, S-CH$_2$-(*Ar*)C-CH-CH-C-C(C$\underline{H_3}$)$_3$), 0.94 (6H, d, (*Ar*)C-CH-CH-C-CH(C$\underline{H_3}$)$_2$, $^3J_{H,H}$ = 6.8 Hz), 0.89 (6H, d, (*Ar*)C-CH-CH-C-CH(C$\underline{H_3}$)$_2$, $^3J_{H,H}$ = 6.8 Hz).

^{13}C-NMR (CDCl$_3$) δ_C, ppm: 171.4 (1C, ($\underline{C}$=O)-(CH$_2$)$_3$-C≡CH), 151.9, 151.8 (2C, 2xS-CH$_2$-(*Ar*)C-CH-CH-$\underline{C}$-C(CH$_3$)$_3$), 151.1 (1C, S-(*Ar*)C-CH-CH-$\underline{C}$-O), 136.8 (2C, 2xS-CH$_2$-(*Ar*)$\underline{C}$-CH-CH-C-C(CH$_3$)$_3$), 135.2 (1C, S-(*Ar*)$\underline{C}$-CH-CH-C-O), 133.7 (2C, 2xS-(*Ar*)C-$\underline{C}$H-CH-C-O), 129.4, 129.2 (4C, 4xS-CH$_2$-(*Ar*)C-$\underline{C}$H-CH-C-C(CH$_3$)$_3$), 125.7, 125.5 (4C, 4xS-CH$_2$-(*Ar*)C-CH-$\underline{C}$H-C-C(CH$_3$)$_3$), 122.4 (2C, 2xS-(*Ar*)C-CH-$\underline{C}$H-C-O), 107.2 (2C, 2xCH$_3$-(*Ar*)C-CH-CH-$\underline{C}$), 100.7 (2C, 2xCH$_3$-(*Ar*)$\underline{C}$-CH-CH-C), 84.3 (2C, 2xCH$_3$-(*Ar*)C-$\underline{C}$H-CH-C), 83.8 (4C, 4xCH$_3$-(*Ar*)C-CH-$\underline{C}$H-C), 83.1 (1C, (C=O)-(CH$_2$)$_3$-$\underline{C}$≡CH), 82.7 (2C, 2xCH$_3$-(*Ar*)C-$\underline{C}$H-CH-C), 69.6 (1C, (C=O)-(CH$_2$)$_2$-C≡$\underline{C}$H), 40.1 (1C, S-$\underline{C}$H$_2$-(*Ar*)C-CH-CH-C-C(CH$_3$)$_3$), 39.7 (1C, S-$\underline{C}$H$_2$-(*Ar*)C-CH-CH-C-C(CH$_3$)$_3$), 34.91, 34.87 (2C, 2xS-CH$_2$-(*Ar*)C-CH-CH-C-$\underline{C}$(CH$_3$)$_3$), 33.0 (1C, (C=O)-$\underline{C}$H$_2$-(CH$_2$)$_2$-C≡CH), 31.55, 31.53 (6C, 2xS-CH$_2$-(*Ar*)C-CH-CH-C-C($\underline{C}$H$_3$)$_3$), 31.0 (2C, 2x(*Ar*)CH-CH-C-$\underline{C}$H(CH$_3$)$_2$), 23.5 (1C, (C=O)-CH$_2$-$\underline{C}$H$_2$-CH$_2$-C≡CH), 23.2 (2C, (*Ar*)CH-CH-C-CH($\underline{C}$H$_3$)$_2$), 22.7 (2C, (*Ar*)CH-CH-C-CH($\underline{C}$H$_3$)$_2$), 18.3 (2C, 2x$\underline{C}$H$_3$-(*Ar*)C-CH-CH), 17.9 (1C, (C=O)-(CH$_2$)$_2$-$\underline{C}$H$_2$-C≡CH).

R$_f$(CH$_2$Cl$_2$/CH$_3$OH 10:1 (v/v)) = 0.452.

ESI-MS(+): *m*/*z* found 1049.2544 [M-Cl]$^+$, calcd. for $C_{54}H_{70}O_2Ru_2S_3^+$ 1049.2541.

Elemental analysis (%): calcd. for $C_{54}H_{69}ClO_2Ru_2S_3$ C 59.84, H 6.42; found C 59.49, H 6.46.

3. Synthesis of the azide and alkyne functionalized carbohydrate intermediates 10-18

The azide functionalized carbohydrate derivative **10** was prepared and purified by adapting the literature procedure [7,8].

Synthesis of (2*R*,3*R*,4*S*,5*R*,6*R*)-2-(acetoxymethyl)-6-azidotetrahydro-2*H*-pyran-3,4,5-triyl triacetate (10)

To a solution of (2*R*,3*R*,4*S*,5*R*,6*S*)-2-(acetoxymethyl)-6-bromotetrahydro-2*H*-pyran-3,4,5-triyl triacetate (0.500 g, 1.220 mmol, 1 equiv.) in THF (15 mL) were successively added at r.t. azidotrimethylsilane (0.190 g, 1.647 mmol, 1.35 equiv.) and TBAF (1.60 mL, 1.600 mmol, 1.3 equiv.). The reaction mixture was further stirred at r.t. for 4 h, then the mixture was filtered through a plug of silica gel, and the filtrate was concentrated under reduced pressure to dryness. The residue was crystallized from EtOH abs. to afford **10** as a white solid (0.229 mg, 0.616 mmol, yield 51%).

^{1}H-NMR ($CDCl_3$) δ_H, ppm: 5.22 (1H, t, N_3-CH-CH-C<u>*H*</u>-OAc, $^{3}J_{H,H}$ = 9.4 Hz), 5.10 (1H, t, N_3-CH-CH-CH-C<u>*H*</u>-OAc, $^{3}J_{H,H}$ = 9.6 Hz), 4.95 (1H, t, N_3-CH-<u>C</u>H-OAc, $^{3}J_{H,H}$ = 9.5 Hz), 4.64 (1H, d, N_3-C<u>*H*</u>-CH-OAc, $^{3}J_{H,H}$ = 8.8 Hz), 4.27 (1H, dd, CH-C<u>*H*</u>$_2$-OAc, $^{2}J_{H,H}$ = 12.5 Hz, $^{3}J_{H,H}$ = 4.8 Hz), 4.17 (1H, dd, CH-C<u>*H*</u>$_2$-OAc, $^{2}J_{H,H}$ = 12.4 Hz, $^{3}J_{H,H}$ = 2.3 Hz), 3.79 (1H, ddd, C<u>*H*</u>-CH_2-OAc, $^{3}J_{H,H}$ = 10.0 Hz, $^{3}J_{H,H}$ = 4.8 Hz, $^{3}J_{H,H}$ = 2.3 Hz), 2.10 (3H, s, O-(C=O)-C<u>*H*$_3$</u>), 2.07 (3H, s, O-(C=O)-C<u>*H*$_3$</u>), 2.03 (3H, s, O-(C=O)-C<u>*H*$_3$</u>), 2.01 (3H, s, O-(C=O)-C<u>*H*$_3$</u>).

^{13}C-NMR ($CDCl_3$) δ_C, ppm: 170.7 (1C, O-(<u>C</u>=O)-CH_3), 170.3 (1C, O-(<u>C</u>=O)-CH_3), 169.4 (1C, O-(<u>C</u>=O)-CH_3), 169.3 (1C, O-(<u>C</u>=O)-CH_3), 88.1 (1C, N_3-<u>C</u>H-CH-OAc), 74.2 (1C, <u>C</u>H-CH_2-OAc), 72.8 (N_3-CH-CH-<u>C</u>H-OAc), 70.8 (1C, N_3-CH-<u>C</u>H-OAc), 68.0 (1C, <u>C</u>H-CH-CH_2-OAc), 61.8 (1C, CH-<u>C</u>H_2-OAc), 20.8 (1C, O-(C=O)-<u>C</u>H_3), 20.70 (1C, O-(C=O)-<u>C</u>H_3), 20.68 (2C, 2xO-(C=O)-<u>C</u>H_3).

R_f(EtOAc/Hex 2:3 (v/v)) = 0.446.

ESI-MS(+): *m/z* found 391.1449 [M+H_2O]$^+$, 396.0998 [M+Na]$^+$, 746.2064 [2M]$^+$, 769.2112 [2M+Na]$^+$; calcd. for $C_{14}H_{21}N_3O_{10}$ 391.1227, $C_{14}H_{19}N_3NaO_9^+$ 396.1014, $C_{28}H_{38}N_6O_{18}$ 746.2243, $C_{28}H_{38}N_6NaO_{18}^+$ 769.2135.

The bromo **11-13** [9,10] and azide **14-16** [7,8,10] functionalized carbohydrate derivatives were prepared and purified by adapting the literature procedures.

Synthesis of (2*R*,3*R*,4*S*,5*R*,6*R*)-2-(acetoxymethyl)-6-(2-bromoethoxy)tetrahydro-2*H*-pyran-3,4,5-triyl triacetate (11)

To a solution of 1,2,3,4,6-penta-O-acetyl-*β*-D-glucopyranose (1.000 g, 2.560 mmol, 1 equiv.) in dry CH_2Cl_2 (40 mL) at 0°C under inert atmosphere (N_2) were added 2-bromoethanol (0.625 g, 5.120 mmol, 2 equiv.) and $BF_3{\cdot}Et_2O$ (0.508 g, 7.680 mmol, 3 equiv.). The reaction mixture was stirred at r.t. for 24 h and the reaction evolution was verified by TLC. The reaction mixture was concentrated under reduced pressure, then the crude was solubilized in EtOAc (20 mL) and washed with H_2O (20 mL), and the aqueous phase was further extracted with EtOAc (2x20 mL). The combined organic phases were washed with brine (10 mL), dried over anhydrous Na_2SO_4, filtered, and

concentrated under reduced pressure. Purification by column chromatography using a mixture EtOAc/Hex 2:3 (v/v) as eluent afforded **11** as a white solid (0.359 g, 0.790 mmol, yield 32%).

^{1}H-NMR ($CDCl_3$) δ_H, ppm: 5.21 (1H, t, Br-$(CH_2)_2$-O-CH-CH-C$\underline{H}$-OAc, $^3J_{H,H}$ = 9.5 Hz), 5.08 (1H, t, Br-$(CH_2)_2$-O-CH-CH-CH-C$\underline{H}$-OAc, $^3J_{H,H}$ = 9.5 Hz), 5.01 (1H, dd, Br-$(CH_2)_2$-O-CH-C$\underline{H}$-OAc, $^3J_{H,H}$ = 9.6 Hz, $^3J_{H,H}$ = 8.0 Hz), 4.57 (1H, d, Br-$(CH_2)_2$-O-C$\underline{H}$-CH-OAc, $^3J_{H,H}$ = 8.0 Hz), 4.26 (1H, dd, CH-C$\underline{H_2}$-OAc, $^2J_{H,H}$ = 12.3 Hz, $^3J_{H,H}$ = 4.8 Hz), 4.14 (1H, m, CH-C$\underline{H_2}$-OAc, $^2J_{H,H}$ = 12.4 Hz), 4.11-4.19 (1H, m, Br-CH_2-C$\underline{H_2}$-O), 3.78-3.87 (1H, m, Br-CH_2-C$\underline{H_2}$-O), 3.70 (1H, ddd, C$\underline{H}$-CH_2-OAc, $^3J_{H,H}$ = 10.0 Hz, $^3J_{H,H}$ = 4.8 Hz, $^3J_{H,H}$ = 2.4 Hz), 3.42-3.49 (2H, m, O-CH_2-C$\underline{H_2}$-Br), 2.09 (3H, s, O-(C=O)-C$\underline{H_3}$), 2.07 (3H, s, O-(C=O)-C$\underline{H_3}$), 2.02 (3H, s, O-(C=O)-C$\underline{H_3}$), 2.00 (3H, s, O-(C=O)-C$\underline{H_3}$).

^{13}C-NMR ($CDCl_3$) δ_C, ppm: 170.8 (1C, O-($\underline{C}$=O)-CH_3), 170.4 (1C, O-($\underline{C}$=O)-CH_3), 169.6 (2C, 2xO-($\underline{C}$=O)-CH_3), 101.2 (1C, Br-$(CH_2)_2$-O-$\underline{C}$H-CH-OAc), 72.8 (1C, Br-$(CH_2)_2$-O-CH-CH-$\underline{C}$H-OAc), 72.1 (1C, $\underline{C}$H-CH_2-OAc), 71.2 (1C, Br-$(CH_2)_2$-O-CH-$\underline{C}$H-OAc), 69.9 (1C, Br-CH_2-$\underline{C}H_2$-O), 68.5 (1C, Br-$(CH_2)_2$-O-CH-CH-CH-$\underline{C}$H-OAc), 62.0 (1C, CH-$\underline{C}H_2$-OAc), 30.0 (1C, Br-$\underline{C}H_2$-CH_2-O), 20.9 (2C, 2xO-(C=O)-$\underline{C}H_3$), 20.8 (1C, O-(C=O)-$\underline{C}H_3$), 20.7 (1C, O-(C=O)-$\underline{C}H_3$).

R_f(EtOAc/Hex 2:3 (v/v)) = 0.436.

ESI-MS(+): *m/z* found 472.0804 $[M+H_2O]^+$, 477.0353 $[M+Na]^+$, 931.0834 $[2M+Na]^+$, calcd. for $C_{16}H_{25}BrO_{11}$ 472.0580, $C_{16}H_{23}BrNaO_{10}^+$ 477.0367, $C_{32}H_{46}Br_2NaO_{20}^+$ 931.0841.

Synthesis of (2*R*,3*S*,4*S*,5*R*,6*R*)-2-(acetoxymethyl)-6-(2-bromoethoxy)tetrahydro-2*H*-pyran-3,4,5-triyl triacetate (12)

To a solution of 1,2,3,4,6-penta-O-acetyl-β-D-galactopyranose (1.000 g, 2.560 mmol, 1 equiv.) in dry CH_2Cl_2 (40 mL) at 0 °C under inert atmosphere (N_2) were added 2-bromoethanol (0.625 g, 5.120 mmol, 2 equiv.) and $BF_3 \cdot Et_2O$ (0.508 g, 7.680 mmol, 3 equiv.). The mixture was stirred at r.t. for 24 h and the reaction evolution was verified by TLC. The reaction mixture was concentrated under reduced pressure, the crude was solubilized in EtOAc (20 mL) and washed with H_2O (20 mL), and then the aqueous phase was further extracted with EtOAc (2x20 mL). The combined organic phases were washed with brine (10 mL), dried over anhydrous Na_2SO_4, filtered, and concentrated under reduced pressure. Purification by column chromatography using a mixture EtOAc/Hex 2:3 (v/v) as eluent afforded **12** as a white solid (0.466 g, 1.027 mmol, yield 41%).

^{1}H-NMR ($CDCl_3$) δ_C, ppm: 5.39 (1H, dd, Br-$(CH_2)_2$-O-CH-CH-CH-C$\underline{H}$-OAc, $^3J_{H,H}$ = 3.4 Hz, $^3J_{H,H}$ = 0.9 Hz), 5.23 (1H, dd, Br-$(CH_2)_2$-O-CH-C$\underline{H}$-OAc, $^3J_{H,H}$ = 10.5 Hz, $^3J_{H,H}$ = 8.0 Hz), 5.02 (1H, dd, Br-$(CH_2)_2$-O-CH-CH-C$\underline{H}$-OAc, $^3J_{H,H}$ = 10.5 Hz, $^3J_{H,H}$ = 3.4 Hz), 4.53 (1H, d, Br-$(CH_2)_2$-O-C$\underline{H}$-CH-OAc, $^3J_{H,H}$ = 8.0 Hz), 4.09-4.21 (3H, m, CH-C$\underline{H_2}$-OAc, Br-CH_2-C$\underline{H_2}$-O), 3.91 (1H, td, C$\underline{H}$-CH_2-OAc, $^3J_{H,H}$ = 6.6 Hz, $^3J_{H,H}$ = 1.0 Hz), 3.82 (1H, ddd, Br-CH_2-C$\underline{H_2}$-O, $^2J_{H,H}$ = 11.3 Hz, $^3J_{H,H}$ = 7.4 Hz , $^3J_{H,H}$ = 6.3 Hz), 3.48 (1H, d, O-CH_2-C$\underline{H_2}$-Br, $^3J_{H,H}$ = 6.0 Hz), 3.46 (1H, dd, O-CH_2-C$\underline{H_2}$-Br, $^3J_{H,H}$ = 5.8 Hz, $^3J_{H,H}$ = 2.1 Hz), 2.15 (3H, s, O-(C=O)-C$\underline{H_3}$), 2.08 (3H, s, O-(C=O)-C$\underline{H_3}$), 2.05 (3H, s, O-(C=O)-C$\underline{H_3}$), 1.98 (3H, s, O-(C=O)-C$\underline{H_3}$).

^{13}C-NMR ($CDCl_3$) δ_C, ppm: 170.5 (1C, O-($\underline{C}$=O)-CH_3), 170.4 (1C, O-($\underline{C}$=O)-CH_3), 170.3 (1C, O-($\underline{C}$=O)-CH_3), 169.7 (1C, O-($\underline{C}$=O)-CH_3), 101.7 (1C, Br-$(CH_2)_2$-O-$\underline{C}$H-CH-OAc), 70.99 (1C, $\underline{C}$H-CH_2-OAc),

70.90 (1C, Br-$(CH_2)_2$-O-CH-CH-C̲H-OAc), 69.9 (1C, Br-CH_2-C̲H_2-O), 68.7 (1C, Br-$(CH_2)_2$-O-CH-C̲H-OAc), 67.1 (1C, Br-$(CH_2)_2$-O-CH-CH-CH-C̲H-OAc), 61.4 (1C, CH-C̲H_2-OAc), 30.1 (1C, Br-C̲H_2-CH_2-O), 21.0 (1C, O-(C=O)-C̲H_3), 20.82 (1C, O-(C=O)-C̲H_3), 20.80 (1C, O-(C=O)-C̲H_3), 20.7 (1C, O-(C=O)-C̲H_3).

R_f(EtOAc/Hex 2:3 (v/v)) = 0.436.

ESI-MS(+): *m/z* found 472.0804 $[M+H_2O]^+$, 477.0354 $[M+Na]^+$, 931.0835 $[2M+Na]^+$, calcd. for $C_{16}H_{25}BrO_{11}$ 472.0580, $C_{16}H_{23}BrNaO_{10}^+$ 477.0367, $C_{32}H_{46}Br_2NaO_{20}^+$ 931.0841.

Synthesis of (2*R*,3*S*,4*S*,5*R*,6*R*)-2-(acetoxymethyl)-6-(4-bromobutoxy)tetrahydro-2*H*-pyran-3,4,5-triyl triacetate (13)

To a solution of 1,2,3,4,6-penta-O-acetyl-*β*-D-galactopyranose (1.000 g, 2.560 mmol, 1 equiv.) in dry CH_2Cl_2 (40 mL) at 0 °C under inert atmosphere (N_2) were added 4-bromo-1-butanol (0.765 g, 5.120 mmol, 2 equiv.) and $BF_3{\cdot}Et_2O$ (0.508 g, 7.680 mmol, 3 equiv.). The mixture was stirred at r.t. for 24 h and the reaction evolution was verified by TLC. The reaction mixture was concentrated under reduced pressure, then the crude was solubilized in EtOAc (20 mL) and washed with H_2O (20 mL), and the aqueous phase was further extracted with EtOAc (2x20 mL). The combined organic phases were washed with brine (10 mL), dried over anhydrous Na_2SO_4, and then concentrated under reduced pressure. Purification by column chromatography using a mixture EtOAc/Hex 2:3 (v/v) as eluent afforded **13** as a white solid (0.565 g, 1.173 mmol, yield 47%).

^{1}H-NMR ($CDCl_3$) δ_H, ppm: 5.43 (1H, dd, Br-$(CH_2)_4$-O-CH-CH-CH-C*H̲*-OAc, $^3J_{H,H}$ = 3.3 Hz, $^3J_{H,H}$ = 1.3 Hz), 5.28-5.34 (1H, m, Br-$(CH_2)_4$-O-CH-C*H̲*-OAc, $^3J_{H,H}$ = 10.4 Hz, $^3J_{H,H}$ = 2.0 Hz), 5.07-5.12 (2H, m, Br-$(CH_2)_4$-O-CH-CH-C*H̲*-OAc, Br-$(CH_2)_4$-O-C*H̲*-CH-OAc, $^3J_{H,H}$ = 3.7 Hz), 4.16-4.20 (1H, m, C*H̲*-CH_2-OAc, $^3J_{H,H}$ = 6.1 Hz, $^3J_{H,H}$ = 1.0 Hz), 4.10 (1H, dd, CH-C*H̲*$_2$-OAc, $^2J_{H,H}$ = 14.32 Hz, $^3J_{H,H}$ = 7.2 Hz), 4.07 (1H, d, CH-C*H̲*$_2$-OAc, $^3J_{H,H}$ = 6.5 Hz), 3.73 (1H, dt, Br-$(CH_2)_3$-C*H̲*$_2$-O, $^2J_{H,H}$ = 10.0 Hz, $^3J_{H,H}$ = 6.0 Hz), 3.40-3.47 (3H, m, Br-$(CH_2)_3$-C*H̲*$_2$-O, Br-C*H̲*$_2$-$(CH_2)_3$-O, $^3J_{H,H}$ = 6.7 Hz, $^3J_{H,H}$ = 1.5 Hz), 2.12 (3H, s, O-(C=O)-C*H̲*$_3$), 2.06 (3H, s, O-(C=O)-C*H̲*$_3$), 2.03 (3H, s, O-(C=O)-C*H̲*$_3$), 1.97 (3H, s, O-(C=O)-C*H̲*$_3$), 1.90-1.99 (2H, m, Br-CH_2-C*H̲*$_2$-$(CH_2)_2$-O, $^3J_{H,H}$ = 6.8 Hz), 1.70-1.79 (2H, m, Br-CH_2-CH_2-C*H̲*$_2$-CH_2-O, $^3J_{H,H}$ = 6.0 Hz).

R_f(EtOAc/Hex 2:3 (v/v)) = 0.464.

ESI-MS(+): *m/z* found 481.0285 $[M-H]^+$, 500.1117 $[M+H_2O]^+$, 505.0671 $[2M+Na]^+$, calcd. for $C_{18}H_{26}BrO_{10}^+$ 481.0704, $C_{18}H_{29}BrO_{11}$ 500.0893, $C_{18}H_{27}NaO_{10}^+$ 505.0680.

Synthesis of (2*R*,3*R*,4*S*,5*R*,6*R*)-2-(acetoxymethyl)-6-(2-azidoethoxy)tetrahydro-2*H*-pyran-3,4,5-triyl triacetate (14)

To a solution of **11** (0.300 g, 0.658 mmol, 1 equiv.) in dry DMF (10 mL) at r.t. under inert atmosphere (N_2) was added NaN_3 (0.214 g, 3.290 mmol, 5 equiv.). The reaction mixture was stirred at 70°C for 24 h, then was filtered and the filtrate was concentrated under reduced pressure. The residue was solubilized in EtOAc (20 mL) and washed with H_2O (20 mL), and the aqueous phase

was further extracted with EtOAc (2x20 mL). The combined organic phases were washed with brine (20 mL), dried over anhydrous Na_2SO_4, filtered, and concentrated under reduced pressure to afford **14** as a brown liquid (0.130 g, 0.312 mmol, yield 47%).

^{1}H-NMR (CDCl$_3$) δ_H, ppm: 5.20 (1H, t, N_3-(CH_2)$_2$-O-CH-CH-C$\underline{H}$-OAc, $^3J_{H,H}$ = 9.5 Hz), 5.08 (1H, t, N_3-(CH_2)$_2$-O-CH-CH-CH-$\underline{C}$H-OAc, $^3J_{H,H}$ = 9.7 Hz), 5.01 (1H, dd, N_3-(CH_2)$_2$-O-CH-$\underline{C}$H-OAc, $^3J_{H,H}$ = 8.0 Hz, $^3J_{H,H}$ = 9.6 Hz), 4.59 (1H, d, N_3-(CH_2)$_2$-O-C$\underline{H}$-CH-OAc, $^3J_{H,H}$ = 8.0 Hz), 4.24 (1H, dd, CH-C$\underline{H}_2$-OAc, $^2J_{H,H}$ = 12.3 Hz, $^3J_{H,H}$ = 4.7 Hz), 4.15 (1H, dd, CH-C$\underline{H}_2$-OAc, $^2J_{H,H}$ = 12.4 Hz, $^3J_{H,H}$ = 2.4 Hz), 3.99-4.05 (1H, ddd, N_3-CH_2-C$\underline{H}_2$-O, $^2J_{H,H}$ = 10.6 Hz, $^3J_{H,H}$ = 4.8 Hz, $^3J_{H,H}$ = 3.7 Hz), 3.65-3.74 (2H, m, C$\underline{H}$-CH_2-OAc, N_3-CH_2-C$\underline{H}_2$-O), 3.44-3.52 (1H, ddd, O-CH_2-C$\underline{H}_2$-N_3, $^2J_{H,H}$ = 13.4 Hz, $^3J_{H,H}$ = 8.3 Hz, $^3J_{H,H}$ = 3.4 Hz), 3.24-3.31 (1H, ddd, O-CH_2-C$\underline{H}_2$-N_3, $^2J_{H,H}$ = 13.4 Hz, $^3J_{H,H}$ = 4.7 Hz, $^3J_{H,H}$ = 3.4 Hz), 2.07 (3H, s, O-(C=O)-C$\underline{H}_3$), 2.04 (3H, s, O-(C=O)-C$\underline{H}_3$), 2.01 (3H, s, O-(C=O)-C$\underline{H}_3$), 1.99 (3H, s, O-(C=O)-C$\underline{H}_3$).

R$_f$(EtOAc/Hex 2:3 (v/v)) = 0.418.

ESI-MS(+): *m/z* found 435.1713 [M+H_2O]$^+$, 440.1263 [M+Na]$^+$, 852.3102 [2M+H_2O]$^+$, 857.2650 [2M+Na]$^+$, calcd. for $C_{16}H_{25}N_3O_{11}$ 435.1484, $C_{16}H_{23}N_3NaO_{10}^+$ 440.1276, $C_{32}H_{48}N_6O_{20}$ 852.2876, $C_{32}H_{46}N_6NaO_{20}^+$ 857.2659.

Synthesis of (2*R*,3*S*,4*S*,5*R*,6*R*)-2-(acetoxymethyl)-6-(2-azidoethoxy)tetrahydro-2*H*-pyran-3,4,5-triyl triacetate (15)

To a solution of **12** (0.300 g, 0.658 mmol, 1 equiv.) in dry DMF (10 mL) at r.t. under inert atmosphere (N_2) was added NaN_3 (0.214 g, 3.290 mmol, 5 equiv.). The reaction mixture was stirred at 70°C for 24 h, then it was filtered, and the filtrate was concentrated under reduced pressure. The residue was solubilized in EtOAc (20 mL) and washed with H_2O (20 mL), and the aqueous phase was further extracted with EtOAc (2x20 mL). The combined organic phases were washed with brine (10 mL), dried over anhydrous Na_2SO_4, filtered, and then concentrated under reduced pressure to afford **15** as a brown liquid (0.202 g, 0.485 mmol, yield 74%).

^{1}H-NMR (CDCl$_3$) δ_H, ppm: 5.39 (1H, dd, N_3-(CH_2)$_2$-O-CH-CH-CH-C$\underline{H}$-OAc, $^3J_{H,H}$ = 3.4 Hz, $^3J_{H,H}$ = 0.7 Hz), 5.24 (1H, dd, N_3-(CH_2)$_2$-O-CH-C$\underline{H}$-OAc, $^3J_{H,H}$ = 10.5 Hz, $^3J_{H,H}$ = 8.0 Hz), 5.02 (1H, dd, N_3-(CH_2)$_2$-O-CH-CH-C$\underline{H}$-OAc, $^3J_{H,H}$ = 10.5 Hz, $^3J_{H,H}$ = 3.4 Hz), 4.56 (1H, d, N_3-(CH_2)$_2$-O-C$\underline{H}$-CH-OAc, $^3J_{H,H}$ = 8.0 Hz), 4.18 (1H, dd, CH-C$\underline{H}_2$-OAc, $^2J_{H,H}$ = 11.3 Hz, $^3J_{H,H}$ = 6.6 Hz), 4.12 (1H, dd, CH-C$\underline{H}_2$-OAc, $^2J_{H,H}$ = 11.3 Hz, $^3J_{H,H}$ = 6.7 Hz), 4.04 (1H, ddd, O-C$\underline{H}_2$-CH_2-N_3, $^2J_{H,H}$ = 10.7 Hz, $^3J_{H,H}$ = 4.7 Hz, $^3J_{H,H}$ = 3.5 Hz), 3.92 (1H, td, C$\underline{H}$-CH_2-OAc, $^3J_{H,H}$ = 6.6 Hz, $^3J_{H,H}$ = 1.0 Hz), 3.69 (1H, ddd, O-C$\underline{H}_2$-CH_2-N_3, $^2J_{H,H}$ = 10.9 Hz, $^3J_{H,H}$ = 8.5 Hz, $^3J_{H,H}$ = 3.4 Hz), 3.50 (1H, ddd, O-CH_2-C$\underline{H}_2$-N_3, $^2J_{H,H}$ = 13.3 Hz, $^3J_{H,H}$ = 8.6 Hz, $^3J_{H,H}$ = 3.5 Hz), 3.30 (1H, ddd, O-CH_2-C$\underline{H}_2$-N_3, $^2J_{H,H}$ = 13.4 Hz, $^3J_{H,H}$ = 4.5 Hz, $^3J_{H,H}$ = 3.5 Hz), 2.15 (3H, s, O-(C=O)-C$\underline{H}_3$), 2.08 (3H, s, O-(C=O)-C$\underline{H}_3$), 2.05 (3H, s, O-(C=O)-C$\underline{H}_3$), 1.98 (3H, s, O-(C=O)-C$\underline{H}_3$).

R$_f$(EtOAc/Hex 2:3 (v/v)) = 0.400.

ESI-MS(+): *m/z* found 435.1719 [M+H_2O]$^+$, 440.1266 [M+Na]$^+$, 857.2654 [2M+Na]$^+$, calcd. for $C_{16}H_{25}N_3O_{11}$ 435.1489, $C_{14}H_{19}O_9^+$ $C_{16}H_{23}N_3NaO_{10}^+$ 440.1276, $C_{32}H_{46}N_6NaO_{20}^+$ 857.2659.

Synthesis of (2*R*,3*S*,4S,5R,6R)-2-(acetoxymethyl)-6-(4-azidobutoxy)tetrahydro-2*H*-pyran-3,4,5-triyl triacetate (16)

To a solution of **13** (0.240 g, 0.496 mmol, 1 equiv.) in dry DMF (10 mL) at r.t. under inert atmosphere (N_2) was added NaN_3 (0.161 g, 1.480 mmol, 5 equiv.). The reaction mixture was stirred at 70 °C for 24 h, then it was filtered and the filtrate was concentrated under reduced pressure. The residue was solubilized in EtOAc (20 mL), washed with H_2O (20 mL), and the aqueous phase was further extracted with EtOAc (2×20 mL). The organic phases were combined, washed with brine (10 mL), dried over anhydrous Na_2SO_4, filtered, and concentrated under reduced pressure to afford **16** as a brown liquid (0.253 g, 0.567 mmol, quant. yield).

^{1}H-NMR ($CDCl_3$) δ_H, ppm: 5.42 (1H, dd, N_3-$(CH_2)_4$-O-CH-CH-CH-C$\underline{H}$-OAc, $^3J_{H,H}$ = 3.4 Hz, $^3J_{H,H}$ = 1.2 Hz), 5.28-5.34 (1H, m, N_3-$(CH_2)_4$-O-CH-C$\underline{H}$-OAc), 5.06-5.12 (2H, m, N_3-$(CH_2)_4$-O-CH-CH-C$\underline{H}$-OAc, N_3-$(CH_2)_4$-O-C$\underline{H}$-CH-OAc, $^3J_{H,H}$ = 3.7 Hz), 4.15-4.20 (1H, m, C$\underline{H}$-CH_2-OAc, $^3J_{H,H}$ = 6.2 Hz, $^3J_{H,H}$ = 0.9 Hz), 4.07 (2H, d, CH-C$\underline{H_2}$-OAc, $^3J_{H,H}$ = 6.6 Hz), 3.68-3.75 (1H, m, N_3-$(CH_2)_3$-C$\underline{H_2}$-O), 3.38-3.46 (1H, m, N_3-$(CH_2)_3$-C$\underline{H_2}$-O), 3.28-3.33 (2H, m, N_3-C$\underline{H_2}$-$(CH_2)_3$-O), 2.12 (3H, s, O-(C=O)-C$\underline{H_3}$), 2.05 (3H, s, O-(C=O)-C$\underline{H_3}$), 2.02 (3H, s, O-(C=O)-C$\underline{H_3}$), 1.96 (3H, s, O-(C=O)-C$\underline{H_3}$), 1.64-1.69 (4H, m, N_3-CH_2-C$\underline{H_2}$-$(CH_2)_2$-O, N_3-$(CH_2)_2$-C$\underline{H_2}$-CH_2-O, $^3J_{H,H}$ = 3.3 Hz).

^{13}C-NMR ($CDCl_3$) δ_C, ppm: 170.49 (1C, O-($\underline{C}$=O)-CH_3), 170.46 (1C, O-($\underline{C}$=O)-CH_3), 170.3 (1C, O-($\underline{C}$=O)-CH_3), 170.1 (1C, O-($\underline{C}$=O)-CH_3), 96.3 (1C, N_3-$(CH_2)_4$-O-$\underline{C}$H-CH-OAc), 68.3 (1C, N_3-$(CH_2)_4$-O-CH-CH-$\underline{C}$H-OAc), 68.2 (1C, N_3-$(CH_2)_4$-O-CH-CH-CH-$\underline{C}$H-OAc), 68.0 (1C, N_3-$(CH_2)_3$-$\underline{C}H_2$-O), 67.7 (1C, N_3-$(CH_2)_4$-O-CH-$\underline{C}$H-OAc), 66.4 (1C, $\underline{C}$H-CH_2-OAc), 61.9 (1C, CH-$\underline{C}H_2$-OAc), 51.2 (1C, N_3-$\underline{C}H_2$-$(CH_2)_3$-O), 26.6 (1C, N_3-$(CH_2)_2$-$\underline{C}H_2$-CH_2-O), 25.8 (1C, N_3-CH_2-$\underline{C}H_2$-$(CH_2)_2$-O), 20.83 (1C, O-(C=O)-$\underline{C}H_3$), 20.76 (1C, O-(C=O)-$\underline{C}H_3$), 20.74 (1C, O-(C=O)-$\underline{C}H_3$), 20.71 (1C, O-(C=O)-$\underline{C}H_3$).

R_f(EtOAc/Hex 2:3 (v/v)) = 0.491.

ESI-MS(+): *m/z* found 463.2036 $[M+H_2O]^+$, 468.1586 $[M+Na]^+$, 913.3295 $[2M+Na]^+$, calcd. for $C_{18}H_{29}N_3O_{11}$ 463.1802, $C_{18}H_{27}N_3NaO_{10}^+$ 468.1589, $C_{36}H_{54}N_6NaO_{20}^+$ 913.3285.

The alkyne functionalized carbohydrate derivatives **17** and **18** were prepared and purified by adapting a literature procedure [11].

Synthesis of (2*R*,3*R*,4*S*,5*R*,6*R*)-2-(acetoxymethyl)-6-(pent-4-yn-1-yloxy)tetrahydro-2*H*-pyran-3,4,5-triyl triacetate (17)

To a solution of 1,2,3,4,6-penta-O-acetyl-*β*-D-glucopyranose (1.000 g, 2.560 mmol, 1 equiv.) in dry CH_2Cl_2 (20 mL) at 0 °C under inert atmosphere (N_2) were added 4-pentyn-1-ol (0.253 g, 3.070 mmol, 1.2 equiv.) and $BF_3{\cdot}Et_2O$ (0.727 g, 5.120 mmol, 2 equiv.). The reaction mixture was stirred at r.t. for 2 h. Then anhydrous K_2CO_3 (0.48 g) was added and the reaction mixture was further stirred at r.t. for 1 h. The suspension was filtered, and the filtrate was washed with H_2O (2x20 mL). The unified aqueous phases were extracted with CH_2Cl_2 (2x20 mL). The organic phases were combined, washed with brine (10 mL), dried over anhydrous Na_2SO_4, filtered, and concentrated under reduced

pressure. Washing of the crude using a CH_2Cl_2/Hex mixture afforded **17** as a brown liquid (0.686 g, 1.643 mmol, yield 64%).

^{1}H-NMR ($CDCl_3$) δ_H, ppm: 5.19 (1H, t, HC≡C-(CH_2)$_3$-O-CH-CH-C<u>*H*</u>-OAc, $^3J_{H,H}$ = 9.5 Hz), 5.06 (1H, t, HC≡C-(CH_2)$_3$-O-CH-CH-CH-C<u>*H*</u>-OAc, $^3J_{H,H}$ = 9.5 Hz), 4.97 (1H, dd, HC≡C-(CH_2)$_3$-O-CH-C<u>*H*</u>-OAc, $^3J_{H,H}$ = 8.0 Hz, $^3J_{H,H}$ = 9.5 Hz), 4.49 (1H, d, HC≡C-(CH_2)$_3$-O-C<u>*H*</u>-CH-OAc, $^3J_{H,H}$ = 8.0 Hz),
4.25 (1H, dd, CH-C<u>*H*</u>$_2$-OAc, $^2J_{H,H}$ = 12.1 Hz, $^3J_{H,H}$ = 4.7 Hz), 4.12 (1H, dd, CH-C<u>*H*</u>$_2$-OAc, $^2J_{H,H}$ = 12.2 Hz, $^3J_{H,H}$ = 2.5 Hz), 3.94 (1H, dt, HC≡C-(CH_2)$_2$-C<u>*H*</u>$_2$-O, $^2J_{H,H}$ = 9.7 Hz, $^3J_{H,H}$ = 5.5 Hz), 3.68 (1H, ddd, C<u>*H*</u>-CH_2-OAc, $^2J_{H,H}$ = 9.9 Hz, $^3J_{H,H}$ = 5.0 Hz, $^3J_{H,H}$ = 2.5 Hz), 3.57-3.65 (1H, m, HC≡C-(CH_2)$_2$-C<u>*H*</u>$_2$-O, $^3J_{H,H}$ = 5.2 Hz), 2.20-2.28 (2H, m, HC≡C-C<u>*H*</u>$_2$-(CH_2)$_2$-O, $^3J_{H,H}$ = 6.6 Hz, $^3J_{H,H}$ = 2.7 Hz), 2.04 (3H, s, O-(C=O)-C<u>*H*</u>$_3$), 2.02 (3H, s, O-(C=O)-C<u>*H*</u>$_3$), 2.01 (3H, s, O-(C=O)-C<u>*H*</u>$_3$), 1.99 (3H, s, O-(C=O)-C<u>*H*</u>$_3$), 1.93 (1H, t, <u>*H*</u>C≡C-(CH_2)$_3$-O, $^3J_{H,H}$ = 2.7 Hz), 1.69-1.89 (2H, m, HC≡C-CH_2-C<u>*H*</u>$_2$-CH_2-O, $^3J_{H,H}$ = 7.3 Hz).

R_f(EtOAc/Hex 2:3 (v/v)) = 0.393.

ESI-MS(+): *m/z* found 437.1410 [M+Na]$^+$, 851.2944 [2M+Na]$^+$, calcd. for $C_{19}H_{26}NaO_{10}^+$ 437.1418, $C_{38}H_{52}NaO_{20}^+$ 851.2944.

Synthesis of (2*R*,3*S*,4*S*,5*R*,6*R*)-2-(acetoxymethyl)-6-(pent-4-yn-1-yloxy)tetrahydro-2*H*-pyran-3,4,5-triyl triacetate (18)

To a solution of 1,2,3,4,6-penta-O-acetyl-*β*-D-galactopyranose (1.000 g, 2.560 mmol, 1 equiv.) in dry CH_2Cl_2 (20 mL) at 0°C under inert atmosphere (N_2) were added 4-pentyn-1-ol (0.253 g, 3.070 mmol, 1.2 equiv.) and $BF_3{\cdot}Et_2O$ (0.727 g, 5.120 mmol, 2 equiv.). The reaction mixture was stirred at r.t. for 2 h, then anhydrous K_2CO_3 (0.48 g) was added and the reaction mixture was further stirred at r.t. for 1 h. The suspension was filtered, and the filtrate was washed with H_2O (2 x 20 mL). The unified aqueous phases were extracted with CH_2Cl_2 (2 x 10 mL). The organic phases were combined, washed with brine (10 mL), dried over anhydrous Na_2SO_4, filtered, and concentrated under reduced pressure. Washing of the crude using a CH_2Cl_2/Hex mixture afforded **18** as a brown liquid (0.495 g, 1.185 mmol, yield 46%).

^{1}H-NMR ($CDCl_3$) δ_H, ppm: 5.31-5.36 (1H, m, HC≡C-(CH_2)$_3$-O-CH-CH-CH-C<u>*H*</u>-OAc), 5.10-5.17 (1H, m, HC≡C-(CH_2)$_3$-O-CH-C<u>*H*</u>-OAc, $^3J_{H,H}$ = 10.4 Hz), 4.94-5.00 (1H, m, HC≡C-(CH_2)$_3$-O-CH-CH-C<u>*H*</u>-OAc, $^3J_{H,H}$ = 10.5 Hz), 4.42 (1H, d, HC≡C-(CH_2)$_3$-O-C<u>*H*</u>-CH-OAc, $^3J_{H,H}$ = 7.9 Hz), 4.06-4.16 (2H, m, CH-C<u>*H*</u>$_2$-OAc), 4.00-4.06 (1H, m, <u>*H*</u>C≡C-(CH_2)$_3$-O), 3.89-3.96 (1H, m, HC≡C-(CH_2)$_2$-C<u>*H*</u>$_2$-O), 3.87 (1H, t, C<u>*H*</u>-CH_2-OAc, $^3J_{H,H}$ = 6.7 Hz), 3.54-3.63 (1H, m, HC≡C-(CH_2)$_2$-C<u>*H*</u>$_2$-O), 2.17-2.24 (2H, m, HC≡C-C<u>*H*</u>$_2$-(CH_2)$_2$-O, $^3J_{H,H}$ = 6.5 Hz), 2.09 (3H, s, O-(C=O)-C<u>*H*</u>$_3$), 2.01 (1H, s, O-(C=O)-C<u>*H*</u>$_3$), 1.99 (1H, s, O-(C=O)-C<u>*H*</u>$_3$), 1.92 (1H, s, O-(C=O)-C<u>*H*</u>$_3$), 1.74-1.85 (1H, m, HC≡C-CH_2-C<u>*H*</u>$_2$-CH_2-O, $^3J_{H,H}$ = 6.4 Hz), 1.64-1.74 (1H, m, HC≡C-CH_2-C<u>*H*</u>$_2$-CH_2-O, $^3J_{H,H}$ = 6.2 Hz).

R_f(EtOAc/Hex 2:3 (v/v)) = 0.393.

ESI-MS(+): *m/z* found 432.1851 [M+H_2O]$^+$, 437.1401 [M+Na]$^+$, 851.2927 [2M+Na]$^+$ calcd. for $C_{19}H_{28}O_{11}$ 432.1632, $C_{19}H_{26}NaO_{10}^+$ 437.1418, $C_{38}H_{52}NaO_{20}^+$ 851.2944.

4. Synthesis of the carbohydrate functionalized trithiolato-bridged dinuclear ruthenium(II)-arene complexes 19-26

The carbohydrate functionalized trithiolato Ru(II)-arene complexes were prepared and purified by adapting a literature procedure [6,12].

Synthesis of [(η^6-*p*-$MeC_6H_4Pr^i$)$_2Ru_2$(μ_2-$SCH_2C_6H_4$-*p*-Bu^t)$_2$(μ_2-SC_6H_4-*p*-R)]Cl (R = [2-((4-(1-((2*R*,3*R*,4*S*,5*R*,6*R*)-3,4,5-triacetoxy-6-(acetoxymethyl)tetrahydro-2*H*-pyran-2-yl)-1*H*-1,2,3-triazol-4-yl)butanoyl)oxy)]) (19)

To a solution of **6** (0.200 g, 0.184 mmol, 1 equiv.) in dry DMF (10 mL) at r.t. under inert atmosphere (N_2) were successively added **10** (0.082 g, 0.221 mmol, 1.2 equiv.), $CuSO_4{\cdot}5H_2O$ (0.045 g, 0.184 mmol, 1 equiv.) and sodium ascorbate (0.073 g, 0.368 mmol, 2 equiv.). The reaction mixture was stirred at r.t. for 24 h, then it was diluted with EtOAc (100 mL), washed with H_2O (2x100 mL), and the aqueous phase was further extracted with EtOAc (2x100 mL). The organic phases were combined, washed with brine (100 mL), dried over anhydrous Na_2SO_4, filtered, and concentrated under reduced pressure. Purification by column chromatography using CH_2Cl_2/CH_3OH 10:1 (v/v) as eluent afforded **19** as an orange solid (0.076 g, 0.052 mmol, yield 28%).

^{1}H-NMR ($CDCl_3$) δ_H, ppm: 7.79 (2H, d, 2xS-(*Ar*)C-C$\underline{H}$-CH-C-O-(C=O), $^3J_{H,H}$ = 8.6 Hz), 7.74 (1H, O-(C=O)-(CH_2)$_3$-(*Tr*)C-C$\underline{H}$-N), 7.38-7.50 (8H, m, 4xS-CH_2-(*Ar*)C-CH-C$\underline{H}$-C-C(CH_3)$_3$, 4xS-CH_2-(*Ar*)C-C$\underline{H}$-CH-C-C(CH_3)$_3$), 7.06 (2H, d, 2xS-(*Ar*)C-CH-C$\underline{H}$-C-O-(C=O), $^3J_{H,H}$ = 8.6 Hz), 5.96 (1H, d, (*Tr*)N-C$\underline{H}$-CH-OAc, $^3J_{H,H}$ = 9.3 Hz), 5.51 (1H, t, (*Tr*)N-CH-C$\underline{H}$-OAc, $^3J_{H,H}$ = 9.5 Hz), 5.43 (1H, t, (*Tr*)N-CH-CH-C$\underline{H}$-OAc, $^3J_{H,H}$ = 9.3 Hz), 5.25 (1H, t, (*Tr*)N-CH-CH-CH-C$\underline{H}$-OAc, $^3J_{H,H}$ = 9.4 Hz), 5.12 (2H, d, 2xCH_3-(*Ar*)C-C$\underline{H}$-CH-C, $^3J_{H,H}$ = 5.7 Hz), 5.00 (2H, d, 2xCH_3-(*Ar*)C-CH-C$\underline{H}$-C, $^3J_{H,H}$ = 5.8 Hz), 4.91 (2H, d, 2xCH_3-(*Ar*)C-CH-C$\underline{H}$-C, $^3J_{H,H}$ = 5.8 Hz), 4.62 (2H, d, 2xCH_3-(*Ar*)C-C$\underline{H}$-CH-C, $^3J_{H,H}$ = 5.8 Hz), 4.31 (1H, dd, CH-C$\underline{H}_2$-OAc, $^2J_{H,H}$ = 12.6 Hz, $^3J_{H,H}$ = 4.8 Hz), 4.15 (1H, dd, CH-C$\underline{H}_2$-OAc, $^2J_{H,H}$ = 12.6 Hz, $^3J_{H,H}$ = 2.0 Hz), 4.07 (1H, ddd, C$\underline{H}$-CH_2-OAc, $^3J_{H,H}$ = 10.1 Hz, $^3J_{H,H}$ = 4.8 Hz, $^3J_{H,H}$ = 2.1 Hz), 3.61 (2H, s, S-C$\underline{H}_2$-(*Ar*)C-CH-CH-C-(CH_3)$_3$), 3.42 (2H, s, S-C$\underline{H}_2$-(*Ar*)C-CH-CH-C-(CH_3)$_3$), 2.87 (2H, t, O-(C=O)-C$\underline{H}_2$-(CH_2)$_2$-(*Tr*)C, $^3J_{H,H}$ = 6.7 Hz), 2.64 (2H, t, O-(C=O)-(CH_2)$_2$-C$\underline{H}_2$-(*Tr*)C, $^3J_{H,H}$ = 7.4 Hz), 2.15 (2H, qvint, O-(C=O)-CH_2-C$\underline{H}_2$-CH_2-(*Tr*)C, $^3J_{H,H}$ = 7.4 Hz), 2.08 (3H, s, O-(C=O)-C$\underline{H}_3$), 2.06 (3H, s, O-(C=O)-C$\underline{H}_3$), 2.02 (3H, s, O-(C=O)-C$\underline{H}_3$), 1.89 (2H, sept, 2x(*Ar*)C-CH-CH-C-C$\underline{H}$(CH_3)$_2$, $^3J_{H,H}$ = 6.9 Hz), 1.87 (3H, s, O-(C=O)-C$\underline{H}_3$), 1.76 (6H, s, 2xC$\underline{H}_3$-(*Ar*)C-CH-CH-C), 1.36 (9H, s, S-CH_2-(*Ar*)C-CH-CH-C-C(C$\underline{H}_3$)$_3$), 1.32 (9H, s, S-CH_2-(*Ar*)C-CH-CH-C-C(C$\underline{H}_3$)$_3$), 0.94 (6H, d, (*Ar*)C-CH-CH-C-CH(C$\underline{H}_3$)$_2$, $^3J_{H,H}$ = 6.9 Hz), 0.89 (6H, d, (*Ar*)C-CH-CH-C-CH(C$\underline{H}_3$)$_2$, $^3J_{H,H}$ = 6.9 Hz).

^{13}C-NMR ($CDCl_3$) δ_C, ppm: 171.5 (1C, O-($\underline{C}$=O)-(CH_2)$_3$-(*Tr*)C), 170.7 (1C, O-($\underline{C}$=O)-CH_3), 170.0 (1C, O-($\underline{C}$=O)-CH_3), 169.5 (1C, O-($\underline{C}$=O)-CH_3), 169.1 (1C, O-($\underline{C}$=O)-CH_3), 151.9, 151.8 (2C, 2xS-CH_2-(*Ar*)C-CH-CH-$\underline{C}$-C(CH_3)$_3$), 151.1 (1C, S-(*Ar*)C-CH-CH-$\underline{C}$-O), 147.7 (1C, O-(C=O)-(CH_2)$_3$-(*Tr*)$\underline{C}$-CH-N), 136.7 (2C, 2xS-CH_2-(*Ar*)$\underline{C}$-CH-CH-C-C(CH_3)$_3$), 135.1 (1C, S-(*Ar*)$\underline{C}$-CH-CH-C-O), 133.7 (2C, 2xS-(*Ar*)C-$\underline{C}$H-CH-C-O), 129.4, 129.2 (4C, 4xS-CH_2-(*Ar*)C-$\underline{C}$H-CH-C-C(CH_3)$_3$), 125.7, 125.5 (4C, 4xS-CH_2-(*Ar*)C-CH-$\underline{C}$H-C-C(CH_3)$_3$), 122.5 (2C, 2xS-(*Ar*)C-CH-$\underline{C}$H-C-O), 120.3 (1C, O-(C=O)-(CH_2)$_3$-(*Tr*)C-$\underline{C}$H-N), 107.2

(2C, 2xCH$_3$-(*Ar*)C-CH-CH-$\underline{C}$), 100.7 (2C, 2xCH$_3$-(*Ar*)$\underline{C}$-CH-CH-C), 85.6 (1C, (*Tr*)N-$\underline{C}$H-CH-OAc), 84.3 (2C, 2xCH$_3$-(*Ar*)C-$\underline{C}$H-CH-C), 83.8 (4C, 4xCH$_3$-(*Ar*)C-CH-$\underline{C}$H-C), 82.6 (2C, 2xCH$_3$-(*Ar*)C-$\underline{C}$H-CH-C), 75.1 (1C, $\underline{C}$H-CH$_2$-OAc), 73.0 (1C, (*Tr*)N-CH-CH-$\underline{C}$H-OAc), 70.4 (1C, (*Tr*)N-CH-$\underline{C}$H-OAc), 67.9 (1C, (*Tr*)N-CH-CH-CH-$\underline{C}$H-OAc), 61.7 (1C, CH-$\underline{C}$H$_2$-OAc), 40.1 (1C, S-$\underline{C}$H$_2$-(*Ar*)C-CH-CH-C-C(CH$_3$)$_3$), 39.7 (1C, S-$\underline{C}$H$_2$-(*Ar*)C-CH-CH-C-C(CH$_3$)$_3$), 34.91 (1C, S-CH$_2$-(*Ar*)C-CH-CH-C-$\underline{C}$(CH$_3$)$_3$), 34.87 (1C, S-CH$_2$-(*Ar*)C-CH-CH-C-$\underline{C}$(CH$_3$)$_3$), 33.5 (1C, O-(C=O)-(CH$_2$)$_2$-$\underline{C}$H$_2$-(*Tr*)C), 31.55 (3C, S-CH$_2$-(*Ar*)C-CH-CH-C-C($\underline{C}$H$_3$)$_3$), 31.52 (3C, S-CH$_2$-(*Ar*)C-CH-CH-C-C($\underline{C}$H$_3$)$_3$), 31.0 (2C, 2xCH$_3$-(*Ar*)C-CH-CH-C-$\underline{C}$H(CH$_3$)$_2$), 24.9 (1C, O-(C=O)-$\underline{C}$H$_2$-(CH$_2$)$_2$-(*Tr*)C), 24.4 (1C, O-(C=O)-(CH$_2$)$_2$-$\underline{C}$H$_2$-(*Tr*)C), 23.2 (2C, (*Ar*)CH-CH-C-CH-($\underline{C}$H$_3$)$_2$), 22.7 (2C, (*Ar*)CH-CH-C-CH($\underline{C}$H$_3$)$_2$), 20.9 (1C, O-(C=O)-$\underline{C}$H$_3$), 20.7 (2C, 2xO-(C=O)-$\underline{C}$H$_3$), 20.4 (1C, O-(C=O)-$\underline{C}$H$_3$), 18.3 (2C, 2x$\underline{C}$H$_3$-(*Ar*)C-CH-CH).

R*$_f$*(CH$_2$Cl$_2$/CH$_3$OH 10:1 (v/v)) = 0.526.

ESI-MS(+): *m*/*z* found 1422.3648 [M-Cl]$^+$, calcd. for $C_{68}H_{88}N_3O_{11}Ru_2S_3^+$ 1422.3662.

Elemental analysis (%): calcd. for $C_{68}H_{88}ClN_3O_{11}Ru_2S_3 \cdot 0.46CH_2Cl_2$ C 54.95, H 5.99, N 2.81; found C 54.93, H 6.16, N 2.85.

Synthesis of [(η^6-*p*-MeC$_6$H$_4$Pr^i)$_2$Ru$_2$(μ_2-SCH$_2$C$_6$H$_4$-*p*-Bu^t)$_2$(μ_2-SC$_6$H$_4$-*p*-R)]Cl (R = [2-(4-(1-((2*R*,3*R*,4*S*,5*R*,6*R*)-3,4,5-triacetoxy-6-(acetoxymethyl)tetrahydro-2*H*-pyran-2-yl)-1*H*-1,2,3-triazol-4-yl)butanamido)]) (20)

To a solution of **7** (0.220 g, 0.203 mmol, 1 equiv.) in dry DMF (10 mL) at r.t. under inert atmosphere (N$_2$) were successively added **10** (0.090 g, 0.242 mmol, 1.2 equiv.), $CuSO_4 \cdot 5H_2O$ (0.051 g, 0.203 mmol, 1 equiv.) and sodium ascorbate (0.080 g, 0.406 mmol, 2 equiv.). The reaction mixture was stirred at r.t. for 24 h. Then the mixture was diluted with EtOAc (100 mL), washed with H$_2$O (2x100 mL), and the aqueous phase was further extracted with EtOAc (2x100 mL). The organic phases were combined, washed with brine (100 mL), dried over anhydrous Na$_2$SO$_4$, filtered, and concentrated under reduced pressure. Purification by column chromatography using CH$_2$Cl$_2$/CH$_3$OH 10:1 (v/v) as eluent afforded **20** as an orange solid (0.206g, 0.146 mmol, yield 72%).

^{1}H-NMR (CDCl$_3$) δ_H, ppm: 11.17 (1H, N$\underline{H}$-(C=O)-(CH$_2$)$_3$), 8.12 (2H, d, 2xS-(*Ar*)C-CH-C$\underline{H}$-C-NH-(C=O), $^3J_{H,H}$ = 8.2 Hz), 8.04 (1H, NH-(C=O)-(CH$_2$)$_3$-(*Tr*)C-C$\underline{H}$-N), 7.58 (2H, d, 2xS-(*Ar*)C-C$\underline{H}$-CH-C-NH-(C=O), $^3J_{H,H}$ = 8.2 Hz), 7.35-7.52 (8H, m, 4xS-CH$_2$-(*Ar*)C-CH-C$\underline{H}$-C-C(CH$_3$)$_3$, 4xS-CH$_2$-(*Ar*)C-C$\underline{H}$-CH-C-C(CH$_3$)$_3$), 5.90 (1H, d, (*Tr*)N-C$\underline{H}$-CH-OAc, $^3J_{H,H}$ = 9.4 Hz), 5.61 (1H, t, (*Tr*)N-CH-C$\underline{H}$-OAc, $^3J_{H,H}$ = 9.4 Hz), 5.40 (1H, t, (*Tr*)N-CH-CH-C$\underline{H}$-OAc, $^3J_{H,H}$ = 9.4 Hz), 5.27 (1H, t, (*Tr*)N-CH-CH-CH-C$\underline{H}$-OAc, $^3J_{H,H}$ = 9.3 Hz), 4.98 (2H, d, 2xCH$_3$-(*Ar*)C-C$\underline{H}$-CH-C, $^3J_{H,H}$ = 5.4 Hz), 4.88 (2H, d, 2xCH$_3$-(*Ar*)C-CH-C$\underline{H}$-C, $^3J_{H,H}$ = 5.6 Hz), 4.69 (2H, d, 2xCH$_3$-(*Ar*)C-CH-C$\underline{H}$-C, $^3J_{H,H}$ = 5.5 Hz), 4.61 (2H, d, 2xCH$_3$-(*Ar*)C-C$\underline{H}$-CH-C, $^3J_{H,H}$ = 5.5 Hz), 4.29 (1H, dd, CH-C$\underline{H}_2$-OAc, $^2J_{H,H}$ = 12.5 Hz, $^3J_{H,H}$ = 4.6 Hz), 4.16 (1H, dd, CH-C$\underline{H}_2$-OAc, $^2J_{H,H}$ = 12.4 Hz, $^3J_{H,H}$ = 1.2 Hz), 4.01 (1H, ddd, C$\underline{H}$-CH$_2$-OAc, $^3J_{H,H}$ = 10.0 Hz, $^3J_{H,H}$ = 4.8 Hz, $^3J_{H,H}$ = 2.0 Hz), 3.55 (2H, s, S-C$\underline{H}_2$-(*Ar*)C-CH-CH-C-(CH$_3$)$_3$), 3.36 (2H, s, S-C$\underline{H}_2$-(*Ar*)C-CH-CH-C-(CH$_3$)$_3$), 2.89 (2H, t, NH-(C=O)-(CH$_2$)$_2$-C$\underline{H}_2$-(*Tr*)C, $^3J_{H,H}$ = 7.4 Hz), 2.77 (2H, t, NH-(C=O)-C$\underline{H}_2$-(CH$_2$)$_2$-(*Tr*)C, $^3J_{H,H}$ = 7.3 Hz), 2.15 (2H, qvint, NH-(C=O)-CH$_2$-C$\underline{H}_2$-CH$_2$-(*Tr*)C, $^3J_{H,H}$ = 7.2 Hz), 2.09 (3H, s, O-

(C=O)-C$\underline{H_3}$), 2.05 (3H, s, O-(C=O)-C$\underline{H_3}$), 2.02 (3H, s, O-(C=O)-C$\underline{H_3}$), 1.95-2.06 (2H, m, 2x(*Ar*)C-CH-CH-C-C$\underline{H}$(CH_3)$_2$), 1.86 (3H, s, O-(C=O)-C$\underline{H_3}$), 1.66 (6H, s, 2xC$\underline{H_3}$-(*Ar*)C-CH-CH-C), 1.37 (9H, s, S-CH_2-(*Ar*)C-CH-CH-C-C(C$\underline{H_3}$)$_3$), 1.34 (9H, s, S-CH_2-(*Ar*)C-CH-CH-C-C(C$\underline{H_3}$)$_3$), 0.99 (6H, d, (*Ar*)C-CH-CH-C-CH(C$\underline{H_3}$)$_2$, $^3J_{H,H}$ = 6.8 Hz), 0.93 (6H, d, (*Ar*)C-CH-CH-C-CH(C$\underline{H_3}$)$_2$, $^3J_{H,H}$ = 6.8 Hz).

^{13}C-NMR ($CDCl_3$) δ_C, ppm: 173.3 (1C, NH-($\underline{C}$=O)-(CH_2)$_3$-(*Tr*)C), 170.9 (1C, O-($\underline{C}$=O)-CH_3), 170.2 (1C, O-($\underline{C}$=O)-CH_3), 169.5 (1C, O-($\underline{C}$=O)-CH_3), 168.9 (1C, O-($\underline{C}$=O)-CH_3), 152.02, 151.99 (2C, 2xS-CH_2-(*Ar*)C-CH-CH-$\underline{C}$-C(CH_3)$_3$), 148.5 (1C, NH-(C=O)-(CH_2)$_3$-(*Tr*)$\underline{C}$-CH-N), 141.5 (1C, S-(*Ar*)C-CH-CH-$\underline{C}$-NH), 136.8, 136.5 (2C, 2xS-CH_2-(*Ar*)$\underline{C}$-CH-CH-C-C(CH_3)$_3$), 132.7 (2C, 2xS-(*Ar*)C-$\underline{C}$H-CH-C-NH), 129.34 (1C, S-(*Ar*)$\underline{C}$-CH-CH-C-NH), 129.31, 129.1 (4C, 4xS-CH_2-(*Ar*)C-$\underline{C}$H-CH-C-C(CH_3)$_3$), 125.71, 125.65 (4C, 4xS-CH_2-(*Ar*)C-CH-$\underline{C}$H-C-C(CH_3)$_3$), 121.1 (1C, NH-(C=O)-(CH_2)$_3$-(*Tr*)C-$\underline{C}$H-N), 120.5 (2C, 2xS-(*Ar*)C-CH-$\underline{C}$H-C-NH), 107.8 (2C, 2xCH_3-(*Ar*)C-CH-CH-$\underline{C}$), 100.1 (2C, 2xCH_3-(*Ar*)$\underline{C}$-CH-CH-C), 85.6 (1C, (*Tr*)N-$\underline{C}$H-CH-OAc), 84.3 (2C, 2xCH_3-(*Ar*)C-CH-$\underline{C}$H-C), 83.9 (2C, 2xCH_3-(*Ar*)C-CH-$\underline{C}$H-C), 83.2 (2C, 2xCH_3-(*Ar*)C-$\underline{C}$H-CH-C), 82.2 (2C, 2xCH_3-(*Ar*)C-$\underline{C}$H-CH-C), 75.0 (1C, $\underline{C}$H-CH_2-OAc), 73.3 (1C, (*Tr*)N-CH-CH-$\underline{C}$H-OAc), 70.3 (1C, (*Tr*)N-CH-$\underline{C}$H-OAc), 68.0 (1C, (*Tr*)N-CH-CH-CH-$\underline{C}$H-OAc), 61.8 (1C, CH-$\underline{C}H_2$-OAc), 39.9 (1C, S-$\underline{C}H_2$-(*Ar*)C-CH-CH-C-C(CH_3)$_3$), 39.2 (1C, S-$\underline{C}H_2$-(*Ar*)C-CH-CH-C-C(CH_3)$_3$), 36.8 (1C, NH-(C=O)-$\underline{C}H_2$-(CH_2)$_2$-(*Tr*)C), 34.93 (1C, S-CH_2-(*Ar*)C-CH-CH-C-$\underline{C}$(CH_3)$_3$), 34.91 (1C, S-CH_2-(*Ar*)C-CH-CH-C-$\underline{C}$(CH_3)$_3$), 31.6 (6C, 2xS-CH_2-(*Ar*)C-CH-CH-C-C($\underline{C}H_3$)$_3$), 31.1 (2C, 2x(*Ar*)CH-CH-C-$\underline{C}$H(CH_3)$_2$), 25.4 (1C, NH-(C=O)-CH_2-$\underline{C}H_2$-CH_2-(*Tr*)C), 25.3 (1C, NH-(C=O)-(CH_2)$_2$-$\underline{C}H_2$-(*Tr*)C), 23.1 (2C, (*Ar*)CH-CH-C-CH($\underline{C}H_3$)$_2$), 23.0 (2C, (*Ar*)CH-CH-C-CH-($\underline{C}H_3$)$_2$), 20.9 (1C, O-(C=O)-$\underline{C}H_3$), 20.7 (2C, 2xO-(C=O)-$\underline{C}H_3$), 20.4 (1C, O-(C=O)-$\underline{C}H_3$), 18.1 (2C, 2x$\underline{C}H_3$-(*Ar*)C-CH-CH).

R_f(CH_2Cl_2/CH_3OH 10:1 (v/v)) = 0.404.

ESI-MS(+): *m/z* found 1421.3807 [M-Cl]$^+$, calcd. for $C_{68}H_{89}N_4O_{10}Ru_2S_3^+$ 1421.3822.

Elemental analysis (%): calcd. for $C_{68}H_{89}ClN_4O_{10}Ru_2S_3 \cdot 0.35CH_2Cl_2$ C 55.25, H 6.08, N 3.87; found C 55.25, H 6.19, N 3.80.

Synthesis of [(η^6-*p*-$MeC_6H_4Pr^i$)$_2$$Ru_2$($\mu_2$-$SCH_2C_6H_4$-*p*-$Bu^t$)$_2$($\mu_2$-$SC_6H_4$-*p*-R)]Cl (R = [2-(4-(1-(2-(((2*R*,3*R*,4*S*,5*R*,6*R*)-3,4,5-triacetoxy-6-(acetoxymethyl)tetrahydro-2*H*-pyran-2-yl)oxy)ethyl)-1*H*-1,2,3-triazol-4-yl)butanamido]) (21)

To a solution of **7** (0.162 g, 0.155 mmol, 1 equiv.) in dry DMF (10 mL) at r.t. under inert atmosphere (N_2) were successively added **14** (0.130 g, 0.311 mmol, 2 equiv.), $CuSO_4 \cdot 5H_2O$ (0.040 g, 0.155 mmol, 1 equiv.) and sodium ascorbate (0.061 g, 0.311 mmol, 2 equiv.). The reaction mixture was stirred at r.t. for 24 h, then it was solubilized with EtOAc (100 mL), washed with H_2O (2x100 mL), and the aqueous phase was further extracted with EtOAc (2x100 mL). The organic phases were combined, washed with brine (100 mL), dried over anhydrous Na_2SO_4, filtered, and concentrated to dryness under reduced pressure. Purification by column chromatography using CH_2Cl_2/CH_3OH 10:1 (v/v) mixture as eluent afforded **21** as an orange solid (0.155 g, 0.103 mmol, yield 66%).

^{1}H-NMR (CDCl$_3$) δ_H, ppm: 11.13 (1H, s br, (*Ar*)C-N$\underline{H}$-(C=O)), 8.11 (2H, d, 2xS-(*Ar*)C-CH-C$\underline{H}$-C-NH-(C=O), $^{3}J_{H,H}$ = 8.6 Hz), 8.02 (1H, s, O-(CH$_2$)$_2$-(*Tr*)N-C$\underline{H}$-C), 7.58 (2H, d, 2xS-(*Ar*)C-C$\underline{H}$-CH-C-NH-(C=O), $^{3}J_{H,H}$ = 8.5 Hz), 7.36-7.53 (8H, m, 4xS-CH$_2$-(*Ar*)C-CH-C$\underline{H}$-C-C(CH$_3$)$_3$, 4xS-CH$_2$-(*Ar*)C-C$\underline{H}$-CH-C-C(CH$_3$)$_3$), 5.18 (1H, t, (*Tr*)N-(CH$_2$)$_2$-O-CH-CH-C$\underline{H}$-OAc, $^{3}J_{H,H}$ = 9.5 Hz), 5.07 (1H, t, (*Tr*)N-(CH$_2$)$_2$-O-CH-CH-CH-C$\underline{H}$-OAc, $^{3}J_{H,H}$ = 9.8 Hz), 4.95-5.02 (1H, m, (*Tr*)N-(CH$_2$)$_2$-O-CH-C$\underline{H}$-OAc), 4.98 (2H, d, 2xCH$_3$-(*Ar*)C-C$\underline{H}$-CH-C, $^{3}J_{H,H}$ = 5.3 Hz), 4.88 (2H, d, 2xCH$_3$-(*Ar*)C-CH-C$\underline{H}$-C, $^{3}J_{H,H}$ = 5.8 Hz), 4.69 (2H, d, 2xCH$_3$-(*Ar*)C-CH-C$\underline{H}$-C, $^{3}J_{H,H}$ = 5.7 Hz), 4.65 (1H, d, (*Tr*)N-(CH$_2$)$_2$-O-C$\underline{H}$-CH-OAc, $^{3}J_{H,H}$ = 8.0 Hz), 4.61 (2H, d, 2xCH$_3$-(*Ar*)C-C$\underline{H}$-CH-C, $^{3}J_{H,H}$ = 5.8 Hz), 4.54-4.60 (2H, m, (*Tr*)N-C$\underline{H_2}$-CH$_2$-O-CH-OAc, $^{3}J_{H,H}$ = 4.9 Hz), 4.28 (1H, dd, CH-C$\underline{H_2}$-OAc, $^{2}J_{H,H}$ = 12.0 Hz, $^{3}J_{H,H}$ = 4.6 Hz), 4.24-4.31 (1H, m, (*Tr*)N-CH$_2$-C$\underline{H_2}$-O-CH-OAc), 4.15 (1H, dd, CH-C$\underline{H_2}$-OAc, $^{2}J_{H,H}$ = 12.2 Hz, $^{3}J_{H,H}$ = 1.9 Hz), 4.11-4.19 (1H, m, (*Tr*)N-CH$_2$-C$\underline{H_2}$-O-CH-CH-OAc), 3.80 (1H, ddd, C$\underline{H}$-CH$_2$-OAc, $^{3}J_{H,H}$ = 10.0 Hz, $^{3}J_{H,H}$ = 4.3 Hz, $^{3}J_{H,H}$ = 2.1 Hz), 3.55 (2H, s, S-C$\underline{H_2}$-(*Ar*)C-CH-CH-C-(CH$_3$)$_3$), 3.35 (2H, s, S-C$\underline{H_2}$-(*Ar*)C-CH-CH-C-(CH$_3$)$_3$), 2.82-2.92 (2H, m, NH-(C=O)-(CH$_2$)$_2$-C$\underline{H_2}$-(*Tr*)C), 2.69-2.79 (2H, m, NH-(C=O)-C$\underline{H_2}$-(CH$_2$)$_2$-(*Tr*)C), 2.10-2.19 (2H, m, NH-(C=O)-CH$_2$-C$\underline{H_2}$-CH$_2$-(*Tr*)C), 2.09 (3H, s, O-(C=O)-C$\underline{H_3}$), 2.00 (3H, s, O-(C=O)-C$\underline{H_3}$), 1.97 (3H, s, O-(C=O)-C$\underline{H_3}$), 1.96 (3H, s, O-(C=O)-C$\underline{H_3}$), 1.95-2.04 (2H, m, 2x(*Ar*)C-CH-CH-C-C$\underline{H}$(CH$_3$)$_2$), 1.66 (6H, s, 2xC$\underline{H_3}$-(*Ar*)C-CH-CH-C), 1.37 (9H, s, S-CH$_2$-(*Ar*)C-CH-CH-C-C(C$\underline{H_3}$)$_3$), 1.34 (9H, s, S-CH$_2$-(*Ar*)C-CH-CH-C-C(C$\underline{H_3}$)$_3$), 0.99 (6H, d, (*Ar*)C-CH-CH-C-CH(C$\underline{H_3}$)$_2$, $^{3}J_{H,H}$ = 6.8 Hz), 0.94 (6H, d, (*Ar*)C-CH-CH-C-CH(C$\underline{H_3}$)$_2$, $^{3}J_{H,H}$ = 6.9 Hz).

^{13}C-NMR (CDCl$_3$) δ_C, ppm: 173.4 (1C, NH-($\underline{C}$=O)-(CH$_2$)$_3$-(*Tr*)C), 170.9 (1C, O-($\underline{C}$=O)-CH$_3$), 170.3 (1C, O-($\underline{C}$=O)-CH$_3$), 169.63 (1C, O-($\underline{C}$=O)-CH$_3$), 169.59 (1C, O-($\underline{C}$=O)-CH$_3$), 152.02, 152.00 (2C, 2xS-CH$_2$-(*Ar*)C-CH-CH-$\underline{C}$-C(CH$_3$)$_3$), 147.7 (NH-(C=O)-(CH$_2$)$_3$-(*Tr*)$\underline{C}$-CH-N), 141.5 (1C, S-(*Ar*)C-CH-CH-$\underline{C}$-NH), 136.8, 136.5 (2C, 2xS-CH$_2$-(*Ar*)$\underline{C}$-CH-CH-C-C(CH$_3$)$_3$), 132.7 (3C, 2xS-(*Ar*)C-$\underline{C}$H-CH-C-NH, S-(*Ar*)$\underline{C}$-CH-CH-C-NH), 129.3, 129.0 (4C, 4xS-CH$_2$-(*Ar*)C-$\underline{C}$H-CH-C-C(CH$_3$)$_3$), 125.70, 125.65 (4C, 4xS-CH$_2$-(*Ar*)C-CH-$\underline{C}$H-C-C(CH$_3$)$_3$), 123.0 (NH-(C=O)-(CH$_2$)$_3$-(*Tr*)C-$\underline{C}$H-N), 120.4 (2C, 2xS-(*Ar*)C-CH-$\underline{C}$H-C-NH), 107.8 (2C, 2xCH$_3$-(*Ar*)C-CH-CH-$\underline{C}$), 100.8 (1C, (*Tr*)N-(CH$_2$)$_2$-O-$\underline{C}$H-CH-OAc), 100.1 (2C, 2xCH$_3$-(*Ar*)$\underline{C}$-CH-CH-C), 84.3 (2C, 2xCH$_3$-(*Ar*)C-CH-$\underline{C}$H-C), 83.9 (2C, 2xCH$_3$-(*Ar*)C-CH-$\underline{C}$H-C), 83.2 (2C, 2xCH$_3$-(*Ar*)C-$\underline{C}$H-CH-C), 82.2 (2C, 2xCH$_3$-(*Ar*)C-$\underline{C}$H-CH-C), 73.0 (1C, (*Tr*)N-(CH$_2$)$_2$-O-CH-CH-$\underline{C}$H-OAc), 71.9 (1C, $\underline{C}$H-CH$_2$-OAc), 71.1 (1C, (*Tr*)N-(CH$_2$)$_2$-O-CH-$\underline{C}$H-OAc), 68.5 (1C, (*Tr*)N-(CH$_2$)$_2$-O-CH-CH-CH-$\underline{C}$H-OAc), 68.1 (1C, (*Tr*)N-CH$_2$-$\underline{C}$H$_2$-O-CH-CH-OAc), 61.9 (1C, CH-$\underline{C}$H$_2$-OAc), 49.7 (1C, (*Tr*)N-$\underline{C}$H$_2$-CH$_2$-O-CH-CH-OAc), 39.9 (1C, S-$\underline{C}$H$_2$-(*Ar*)C-CH-CH-C-C(CH$_3$)$_3$), 39.2 (1C, S-$\underline{C}$H$_2$-(*Ar*)C-CH-CH-C-C(CH$_3$)$_3$), 36.6 (1C, NH-(C=O)-(CH$_2$)$_2$-$\underline{C}$H$_2$-(*Tr*)C), 34.93 (1C, S-CH$_2$-(*Ar*)C-CH-CH-C-$\underline{C}$(CH$_3$)$_3$), 34.90 (1C, S-CH$_2$-(*Ar*)C-CH-CH-C-$\underline{C}$(CH$_3$)$_3$), 31.54 (3C, S-CH$_2$-(*Ar*)C-CH-CH-C-C($\underline{C}$H$_3$)$_3$), 31.53 (3C, S-CH$_2$-(*Ar*)C-CH-CH-C-C($\underline{C}$H$_3$)$_3$), 31.1 (2C, 2x(*Ar*)CH-CH-C-$\underline{C}$H(CH$_3$)$_2$), 25.3 (2C, NH-(C=O)-$\underline{C}$H$_2$-(CH$_2$)$_2$-(*Tr*)C, NH-(C=O)-CH$_2$-$\underline{C}$H$_2$-CH$_2$-(*Tr*)C), 23.1 (2C, (*Ar*)CH-CH-C-CH-($\underline{C}$H$_3$)$_2$), 23.0 (2C, (*Ar*)CH-CH-C-CH($\underline{C}$H$_3$)$_2$), 20.9 (1C, O-(C=O)-$\underline{C}$H$_3$), 20.8 (1C, O-(C=O)-$\underline{C}$H$_3$), 20.7 (2C, 2xO-(C=O)-$\underline{C}$H$_3$), 18.1 (2C, 2x$\underline{C}$H$_3$-(*Ar*)C-CH-CH).

R$_f$(CH$_2$Cl$_2$/CH$_3$OH 10:1 (v/v)) = 0.509.

ESI-MS(+): *m*/*z* found 1465.4104 [M-Cl]$^+$, calcd. for $C_{70}H_{93}N_4O_{11}Ru_2S_3^+$ 1465.4084.

Elemental analysis (%): calcd. for $C_{70}H_{93}ClN_4O_{11}Ru_2S_3{\cdot}0.9CH_2Cl_2$ C 55.48 H, 6.71, N 3.37; found C 55.46, H 6.50, N 3.52.

Synthesis of [(η^6-*p*-MeC$_6$H$_4$Pr^i)$_2$Ru$_2$(μ_2-SCH$_2$C$_6$H$_4$-*p*-Bu^t)$_2$(μ_2-SC$_6$H$_4$-*p*-R)]Cl (R = [2-(4-(1-(2-(((2*R*,3*R*,4*S*,5*S*,6*R*)-3,4,5-triacetoxy-6-(acetoxymethyl)tetrahydro-2*H*-pyran-2-yl)oxy)ethyl)-1*H*-1,2,3-triazol-4-yl)butanamido)] (22)

To a solution of **7** (0.162 g, 0.155 mmol, 1 equiv.) in dry DMF (10 mL) at r.t. under inert atmosphere (N_2) were successively added **15** (0.130 g, 0.311 mmol, 2 equiv.), $CuSO_4{\cdot}5H_2O$ (0.040 g, 0.155 mmol, 1 equiv.) and sodium ascorbate (0.061 g, 0.311 mmol, 2 equiv.). The reaction mixture was stirred at r.t. for 24 h, then it was solubilized in EtOAc (100 mL), washed with H_2O (2x100 mL), and the aqueous phase was further extracted with EtOAc (2x100 mL). The organic phases were combined, washed with brine (100 mL), dried over anhydrous Na_2SO_4, filtered, and concentrated to dryness under reduced pressure. Purification by column chromatography using CH_2Cl_2/CH_3OH 10:1 (v/v) mixture as eluent afforded **22** as an orange solid (0.151 g, 0.100 mmol, yield 65%).

^{1}H-NMR (CDCl$_3$) δ_H, ppm: 11.12 (1H, s br, (*Ar*)C-N<u>*H*</u>-(C=O)), 8.10 (2H, d, 2xS-(*Ar*)C-CH-C<u>*H*</u>-C-NH-(C=O), $^3J_{H,H}$ = 8.5 Hz), 8.02 (1H, s br, O-(CH$_2$)$_2$-(*Tr*)N-C<u>*H*</u>-C), 7.57 (2H, d, 2xS-(*Ar*)C-C<u>*H*</u>-CH-C-NH-(C=O), $^3J_{H,H}$ = 8.3 Hz), 7.36-7.51 (8H, m, 4xS-CH$_2$-(*Ar*)C-CH-C<u>*H*</u>-C-C(CH$_3$)$_3$, 4xS-CH$_2$-(*Ar*)C-C<u>*H*</u>-CH-C-C(CH$_3$)$_3$), 5.38 (1H, d, (*Tr*)N-(CH$_2$)$_2$-O-CH-CH-CH-C<u>*H*</u>-OAc, $^3J_{H,H}$ = 3.0 Hz), 5.18 (1H, dd, (*Tr*)N-(CH$_2$)$_2$-O-CH-C<u>*H*</u>-OAc, $^3J_{H,H}$ = 10.4 Hz, $^3J_{H,H}$ = 8.0 Hz), 4.96-5.02 (1H, m, (*Tr*)N-(CH$_2$)$_2$-O-CH-CH-C<u>*H*</u>-OAc, $^3J_{H,H}$ = 3.4 Hz), 4.98 (2H, d, 2xCH$_3$-(*Ar*)C-C<u>*H*</u>-CH-C, $^3J_{H,H}$ = 5.5 Hz), 4.87 (2H, d, 2xCH$_3$-(*Ar*)C-CH-C<u>*H*</u>-C, $^3J_{H,H}$ = 5.8 Hz), 4.69 (2H, d, 2xCH$_3$-(*Ar*)C-CH-C<u>*H*</u>-C, $^3J_{H,H}$ = 5.7 Hz), 4.63 (1H, d, (*Tr*)N-(CH$_2$)$_2$-O-C<u>*H*</u>-CH-OAc, $^3J_{H,H}$ = 8.0 Hz), 4.60 (2H, d, 2xCH$_3$-(*Ar*)C-C<u>*H*</u>-CH-C, $^3J_{H,H}$ = 5.9 Hz), 4.54-4.59 (2H, m, (*Tr*)N-C<u>*H$_2$*</u>-CH$_2$-O), 4.28 (1H, dt, (*Tr*)N-CH$_2$-C<u>*H$_2$*</u>-O, $^2J_{H,H}$ = 10.9 Hz, $^3J_{H,H}$ = 4.4 Hz), 4.13-4.18 (2H, m, CH-C<u>*H$_2$*</u>-OAc, $^3J_{H,H}$ = 7.5 Hz, $^3J_{H,H}$ = 1.7 Hz), 4.09-4.18 (1H, m, (*Tr*)N-CH$_2$-C<u>*H$_2$*</u>-O), 4.00 (1H, t, C<u>*H*</u>-CH$_2$-OAc, $^3J_{H,H}$ = 6.9 Hz), 3.54 (2H, s, S-C<u>*H$_2$*</u>-(*Ar*)C-CH-CH-C-(CH$_3$)$_3$), 3.35 (2H, s, S-C<u>*H$_2$*</u>-(*Ar*)C-CH-CH-C-(CH$_3$)$_3$), 2.81-2.91 (2H, m, NH-(C=O)-(CH$_2$)$_2$-C<u>*H$_2$*</u>-(*Tr*)C), 2.71-2.78 (2H, m, NH-(C=O)-C<u>*H$_2$*</u>-(CH$_2$)$_2$-(*Tr*)C), 2.09-2.23 (2H, m, NH-(C=O)-CH$_2$-C<u>*H$_2$*</u>-CH$_2$-(*Tr*)C), 2.13 (3H, s, O-(C=O)-C<u>*H$_3$*</u>), 2.04 (3H, s, O-(C=O)-C<u>*H$_3$*</u>), 1.97 (3H, s, O-(C=O)-C<u>*H$_3$*</u>), 1.94 (3H, s, O-(C=O)-C<u>*H$_3$*</u>), 1.92-2.02 (2H, m, 2x(*Ar*)C-CH-CH-C-C<u>*H*</u>(CH$_3$)$_2$), 1.66 (6H, s, 2xC<u>*H$_3$*</u>-(*Ar*)C-CH-CH-C), 1.36 (9H, s, S-CH$_2$-(*Ar*)C-CH-CH-C-C(C<u>*H$_3$*</u>)$_3$), 1.34 (9H, s, S-CH$_2$-(*Ar*)C-CH-CH-C-C(C<u>*H$_3$*</u>)$_3$), 0.98 (6H, d, (*Ar*)C-CH-CH-C-CH(C<u>*H$_3$*</u>)$_2$, $^3J_{H,H}$ = 6.8 Hz), 0.94 (6H, d, (*Ar*)C-CH-CH-C-CH(C<u>*H$_3$*</u>)$_2$, $^3J_{H,H}$ = 6.9 Hz).

^{13}C-NMR (CDCl$_3$) δ_C, ppm: 173.4 (1C, (*Ar*)C-NH-(<u>C</u>=O)), 170.6 (1C, O-(<u>C</u>=O)-CH$_3$), 170.4 (1C, O-(<u>C</u>=O)-CH$_3$), 170.1 (1C, O-(<u>C</u>=O)-CH$_3$), 169.7 (1C, O-(<u>C</u>=O)-CH$_3$), 152.01, 151.98 (2C, 2xS-CH$_2$-(*Ar*)C-CH-CH-<u>C</u>-C(CH$_3$)$_3$), 147.4 (1C, O-(CH$_2$)$_2$-(*Tr*)N-CH-<u>C</u>), 141.5 (1C, S-(*Ar*)C-CH-CH-<u>C</u>-NH), 136.8, 136.5 (2C, 2xS-CH$_2$-(*Ar*)<u>C</u>-CH-CH-C-C(CH$_3$)$_3$), 132.7 (2C, 2xS-(*Ar*)C-<u>C</u>H-CH-C-NH), 129.4 (1C, S-(*Ar*)<u>C</u>-CH-CH-C-NH), 129.3, 129.0 (4C, 4xS-CH$_2$-(*Ar*)C-<u>C</u>H-CH-C-C(CH$_3$)$_3$), 125.7, 125.6 (4C, 4xS-CH$_2$-(*Ar*)C-CH-<u>C</u>H-C-C(CH$_3$)$_3$), 123.2 (1C, O-(CH$_2$)$_2$-(*Tr*)N-<u>C</u>H-C), 120.4 (2C, 2xS-(*Ar*)C-CH-<u>C</u>H-C-NH), 107.8 (2C, 2xCH$_3$-(*Ar*)C-CH-CH-<u>C</u>), 101.1 (1C, (*Tr*)N-(CH$_2$)$_2$-O-<u>C</u>H-CH-OAc), 100.1 (2C,

2xCH_3-(*Ar*)$\underline{C}$-CH-CH-C), 84.3 (2C, 2xCH_3-(*Ar*)C-CH-$\underline{C}$H-C), 83.8 (2C, 2xCH_3-(*Ar*)C-CH-$\underline{C}$H-C), 83.2 (2C, 2xCH_3-(*Ar*)C-$\underline{C}$H-CH-C), 82.2 (2C, 2xCH_3-(*Ar*)C-$\underline{C}$H-CH-C), 71.0 (1C, (*Tr*)N-$(CH_2)_2$-O-CH-CH-$\underline{C}$H-OAc), 70.8 (1C, $\underline{C}$H-CH_2-OAc), 68.6 (1C, (*Tr*)N-$(CH_2)_2$-O-CH-$\underline{C}$H-OAc), 67.9 (1C, (*Tr*)N-CH_2-$\underline{C}H_2$-O), 67.3 (1C, (*Tr*)N-$(CH_2)_2$-O-CH-CH-CH-$\underline{C}$H-OAc), 61.5 (1C, CH-$\underline{C}H_2$-OAc), 49.8 (1C, (*Tr*)N-$\underline{C}H_2$-CH_2-O-CH), 39.9 (1C, S-$\underline{C}H_2$-(*Ar*)C-CH-CH-C-$C(CH_3)_3$), 39.2 (1C, S-$\underline{C}H_2$-(*Ar*)C-CH-CH-C-$C(CH_3)_3$), 36.7 (1C, NH-(C=O)-$\underline{C}H_2$-$(CH_2)_2$-(*Tr*)C), 34.92 (1C, S-CH_2-(*Ar*)C-CH-CH-C-$\underline{C}(CH_3)_3$), 34.89 (1C, S-CH_2-(*Ar*)C-CH-CH-C-$\underline{C}(CH_3)_3$), 31.53 (3C, S-CH_2-(*Ar*)C-CH-CH-C-$C(\underline{C}H_3)_3$), 31.52 (3C, S-CH_2-(*Ar*)C-CH-CH-C-$C(\underline{C}H_3)_3$), 31.1 (2C, 2xCH_3-(*Ar*)C-CH-CH-C-$\underline{C}H(CH_3)_2$), 25.3 (1C, NH-(C=O)-$(CH_2)_2$-$\underline{C}H_2$-(*Tr*)C), 25.2 (1C, NH-(C=O)-CH_2-$\underline{C}H_2$-CH_2-(*Tr*)C), 23.1 (2C, (*Ar*)CH-CH-C-CH-$(\underline{C}H_3)_2$), 23.0 (2C, (*Ar*)CH-CH-C-CH-$(\underline{C}H_3)_2$), 20.88 (1C, O-(C=O)-$\underline{C}H_3$), 20.85 (1C, O-(C=O)-$\underline{C}H_3$), 20.81 (1C, O-(C=O)-$\underline{C}H_3$), 20.7 (1C, O-(C=O)-$\underline{C}H_3$), 18.1 (2C, 2x$\underline{C}H_3$-(*Ar*)C-CH-CH).
R_f(CH_2Cl_2/CH_3OH 10:1 (v/v)) = 0.561.
ESI-MS(+): *m*/*z* found 1465.4096 $[M-Cl]^+$, calcd. for $C_{70}H_{93}N_4O_{11}Ru_2S_3^+$ 1465.4084.
Elemental analysis (%): calcd. for $C_{70}H_{93}ClN_4O_{11}Ru_2S_3 \cdot CH_2Cl_2$ C 55.58, H 6.72, N 3.37; found C 55.57, H 6.45, N 3.57.

Synthesis of $[(\eta^6\text{-}p\text{-}MeC_6H_4Pr^i)_2Ru_2(\mu_2\text{-}SCH_2C_6H_4\text{-}p\text{-}Bu^t)_2(\mu_2\text{-}SC_6H_4\text{-}p\text{-}R)]Cl$ (R = [2-(4-(1-(4-(((2*R*,3*R*,4*S*,5*S*,6*R*)-3,4,5-triacetoxy-6-(acetoxymethyl)tetrahydro-2*H*-pyran-2-yl)oxy)butyl)-1*H*-1,2,3-triazol-4-yl)butanamido)] (23)

To a solution of **7** (0.212 g, 0.202 mmol, 1 equiv.) in dry DMF (10 mL) at r.t., under inert atmosphere (N_2) were successively added **16** (0.180 g, 0.404 mmol, 2 equiv.), $CuSO_4 \cdot 5H_2O$ (0.050 g, 0.202 mmol, 1 equiv.) and sodium ascorbate (0.080 g, 0.404 mmol, 2 equiv.). The reaction mixture was stirred at r.t. for 24 h, then it was solubilized with EtOAc (100 mL), washed with H_2O (2x100 mL), the aqueous phase was further extracted with EtOAc (2x100 mL). The organic phases were combined, washed with brine (100 mL), dried over anhydrous Na_2SO_4, filtered, and concentrated under reduced pressure. Purification by column chromatography using CH_2Cl_2/CH_3OH (10:1 (v/v)) as eluent afforded **23** as an orange solid (0.228 g, 0.149 mmol, yield 74%).
^{1}H-NMR ($CDCl_3$) δ_H, ppm: 11.13 (1H, s br, (*Ar*)C-N$\underline{H}$-(C=O)), 8.10 (2H, d, 2xS-(*Ar*)C-CH-C$\underline{H}$-C-NH-(C=O), $^3J_{H,H}$ = 8.5 Hz), 8.06 (1H, s br, O-$(CH_2)_4$-(*Tr*)N-C$\underline{H}$-C), 7.56 (2H, d, 2xS-(*Ar*)C-C$\underline{H}$-CH-C-NH-(C=O), $^3J_{H,H}$ = 8.3 Hz), 7.36-7.50 (8H, m, 4xS-CH_2-(*Ar*)C-CH-C$\underline{H}$-C-$C(CH_3)_3$, 4xS-CH_2-(*Ar*)C-C$\underline{H}$-CH-C-$C(CH_3)_3$), 5.44 (1H, dd, (*Tr*)N-$(CH_2)_4$-O-CH-CH-CH-C$\underline{H}$-OAc), $^3J_{H,H}$ = 3.5 Hz, $^3J_{H,H}$ = 1.0 Hz), 5.32 (1H, dd, (*Tr*)N-$(CH_2)_4$-O-CH-CH-C$\underline{H}$-OAc, $^3J_{H,H}$ = 10.8 Hz, $^3J_{H,H}$ = 3.4 Hz), 5.12 (1H, dd, (*Tr*)N-$(CH_2)_4$-O-CH-C$\underline{H}$-OAc, $^3J_{H,H}$ = 10.8 Hz, $^3J_{H,H}$ = 3.6 Hz), 5.07 (1H, d, (*Tr*)N-$(CH_2)_4$-O-C$\underline{H}$-CH-OAc, $^3J_{H,H}$ = 3.7 Hz), 4.97 (2H, d, 2xCH_3-(*Ar*)C-C$\underline{H}$-CH-C, $^3J_{H,H}$ = 5.7 Hz), 4.87 (2H, d, 2xCH_3-(*Ar*)C-CH-C$\underline{H}$-C, $^3J_{H,H}$ = 5.8 Hz), 4.69 (2H, d, 2xCH_3-(*Ar*)C-CH-C$\underline{H}$-C, $^3J_{H,H}$ = 5.7 Hz), 4.60 (2H, d, 2xCH_3-(*Ar*)C-C$\underline{H}$-CH-C, $^3J_{H,H}$ = 5.8 Hz), 4.41 (2H, t, (*Tr*)N-C$\underline{H_2}$-$(CH_2)_3$-O, $^3J_{H,H}$ = 6.9 Hz), 4.20 (1H, t, C$\underline{H}$-CH_2-OAc, $^3J_{H,H}$ = 6.7 Hz), 4.08 (1H, m, CH-C$\underline{H_2}$-OAc, $^3J_{H,H}$ = 2.0 Hz), 4.07 (1H, m, CH-C$\underline{H_2}$-OAc, $^3J_{H,H}$ = 3.1 Hz), 3.75 (1H, dt, (*Tr*)N-$(CH_2)_3$-C$\underline{H_2}$-O, $^3J_{H,H}$ = 9.9 Hz, $^3J_{H,H}$ = 6.0 Hz), 3.54 (2H, s, S-C$\underline{H_2}$-(*Ar*)C-CH-CH-C-$(CH_3)_3$), 3.44

(1H, dt, (*Tr*)N-(CH$_2$)$_3$-C$\underline{H_2}$-O, $^3J_{H,H}$ = 9.9 Hz, $^3J_{H,H}$ = 6.4 Hz), 3.35 (2H, s, S-C$\underline{H_2}$-(*Ar*)C-CH-CH-C-(CH$_3$)$_3$), 2.82-2.91 (2H, m br, NH-(C=O)-(CH$_2$)$_2$-C$\underline{H_2}$-(*Tr*)C), 2.70-2.78 (2H, m br, NH-(C=O)-C$\underline{H_2}$-(CH$_2$)$_2$-(*Tr*)C), 2.09-2.20 (2H, m br, NH-(C=O)-CH$_2$-C$\underline{H_2}$-CH$_2$-(*Tr*)C), 2.11 (3H, s, O-(C=O)-C$\underline{H_3}$), 2.06 (3H, s, O-(C=O)-C$\underline{H_3}$), 2.04 (3H, s, O-(C=O)-C$\underline{H_3}$), 1.96 (3H, s, O-(C=O)-C$\underline{H_3}$), 1.93-2.03 (2H, m, 2x(*Ar*)C-CH-CH-C-C$\underline{H}$(CH$_3$)$_2$, $^3J_{H,H}$ = 7.0 Hz), 1.66 (6H, s, 2xC$\underline{H_3}$-(*Ar*)C-CH-CH-C), 1.36 (9H, s, S-CH$_2$-(*Ar*)C-CH-CH-C-C(C$\underline{H_3}$)$_3$), 1.34 (9H, s, S-CH$_2$-(*Ar*)C-CH-CH-C-C(C$\underline{H_3}$)$_3$), 0.98 (6H, d, (*Ar*)C-CH-CH-C-CH(C$\underline{H_3}$)$_2$, $^3J_{H,H}$ = 6.8 Hz), 0.93 (6H, d, (*Ar*)C-CH-CH-C-CH(C$\underline{H_3}$)$_2$, $^3J_{H,H}$ = 6.9 Hz).

^{13}C-NMR (CDCl$_3$) δ_C, ppm: 173.5 (1C, (*Ar*)C-NH-($\underline{C}$=O)), 170.63 (1C, O-($\underline{C}$=O)-CH$_3$), 170.61 (1C, O-($\underline{C}$=O)-CH$_3$), 170.4 (1C, O-($\underline{C}$=O)-CH$_3$), 170.0 (1C, O-($\underline{C}$=O)-CH$_3$), 152.0, 151.97 (2C, 2xS-CH$_2$-(*Ar*)C-CH-CH-$\underline{C}$-C(CH$_3$)$_3$), 143.6 (1C, O-(CH$_2$)$_4$-(*Tr*)N-CH-$\underline{C}$), 141.5 (1C, S-(*Ar*)C-CH-CH-$\underline{C}$-NH), 136.8, 136.5 (2C, 2xS-CH$_2$-(*Ar*)$\underline{C}$-CH-CH-C-C(CH$_3$)$_3$), 132.7 (2C, 2xS-(*Ar*)C-$\underline{C}$H-CH-C-NH), 129.4 (1C, S-(*Ar*)$\underline{C}$-CH-CH-C-NH), 129.3, 129.0 (4C, 4xS-CH$_2$-(*Ar*)C-$\underline{C}$H-CH-C-C(CH$_3$)$_3$), 125.7, 125.6 (4C, 4xS-CH$_2$-(*Ar*)C-CH-$\underline{C}$H-C-C(CH$_3$)$_3$), 122.7 (1C, O-(CH$_2$)$_4$-(*Tr*)N-$\underline{C}$H-C), 120.4 (2C, 2xS-(*Ar*)C-CH-$\underline{C}$H-C-NH), 107.8 (2C, 2xCH$_3$-(*Ar*)C-CH-CH-$\underline{C}$), 100.1 (2C, 2xCH$_3$-(*Ar*)$\underline{C}$-CH-CH-C), 96.4 (1C, (*Tr*)N-(CH$_2$)$_4$-O-$\underline{C}$H-CH-OAc), 84.3 (2C, 2xCH$_3$-(*Ar*)C-CH-$\underline{C}$H-C), 83.8 (2C, 2xCH$_3$-(*Ar*)C-CH-$\underline{C}$H-C), 83.2 (2C, 2xCH$_3$-(*Ar*)C-$\underline{C}$H-CH-C), 82.2 (2C, 2xCH$_3$-(*Ar*)C-$\underline{C}$H-CH-C), 68.3 (1C, (*Tr*)N-(CH$_2$)$_4$-O-CH-CH-CH-$\underline{C}$H-OAc), 68.12 (1C, (*Tr*)N-(CH$_2$)$_4$-O-CH-$\underline{C}$H-OAc), 68.05 (1C, (*Tr*)N-(CH$_2$)$_3$-$\underline{C}$H$_2$-O), 67.9 (1C, (*Tr*)N-(CH$_2$)$_4$-O-CH-CH-$\underline{C}$H-OAc), 66.4 (1C, $\underline{C}$H-CH$_2$-OAc), 62.0 (1C, CH-$\underline{C}$H$_2$-OAc), 49.9 (1C, (*Tr*)N-$\underline{C}$H$_2$-(CH$_2$)$_3$-O), 39.9 (1C, S-$\underline{C}$H$_2$-(*Ar*)C-CH-CH-C-C(CH$_3$)$_3$), 39.2 (1C, S-$\underline{C}$H$_2$-(*Ar*)C-CH-CH-C-C(CH$_3$)$_3$), 36.8 (1C, NH-(C=O)-$\underline{C}$H$_2$-(CH$_2$)$_2$-(*Tr*)C), 34.91 (1C, S-CH$_2$-(*Ar*)C-CH-CH-C-$\underline{C}$(CH$_3$)$_3$), 34.88 (1C, S-CH$_2$-(*Ar*)C-CH-CH-C-$\underline{C}$(CH$_3$)$_3$), 31.53 (3C, S-CH$_2$-(*Ar*)C-CH-CH-C-C($\underline{C}$H$_3$)$_3$), 31.51 (3C, S-CH$_2$-(*Ar*)C-CH-CH-C-C($\underline{C}$H$_3$)$_3$), 31.1 (2C, 2xCH$_3$-(*Ar*)C-CH-CH-C-$\underline{C}$H(CH$_3$)$_2$), 27.2 (1C, (*Tr*)N-(CH$_2$)$_2$-$\underline{C}$H$_2$-CH$_2$-O), 26.5 (1C, (*Tr*)N-CH$_2$-$\underline{C}$H$_2$-(CH$_2$)$_2$-O), 25.4 (2C, NH-(C=O)-(CH$_2$)$_2$-$\underline{C}$H$_2$-(*Tr*)C, NH-(C=O)-CH$_2$-$\underline{C}$H$_2$-CH$_2$-(*Tr*)C), 23.1 (2C, (*Ar*)CH-CH-C-CH($\underline{C}$H$_3$)$_2$), 23.0 (2C, (*Ar*)CH-CH-C-CH($\underline{C}$H$_3$)$_2$), 21.0 (1C, O-(C=O)-$\underline{C}$H$_3$), 20.9 (1C, O-(C=O)-$\underline{C}$H$_3$), 20.8 (2C, 2xO-(C=O)-$\underline{C}$H$_3$), 18.1 (2C, 2x$\underline{C}$H$_3$-(*Ar*)C-CH-CH).

R*f*(CH$_2$Cl$_2$/CH$_3$OH 10:1 (v/v)) = 0.534.

ESI-MS(+): *m/z* found 1493.4407 [M-Cl]$^+$, calcd. for $C_{72}H_{97}N_4O_{11}Ru_2S_3^+$ 1493.4403.

Elemental analysis (%): calcd. for $C_{72}H_{97}ClN_4O_{11}Ru_2S_3\cdot 1.5CH_3OH\cdot 0.3H_2O$ C 55.81, H 6.60, N 3.54; found: C 55.84, H 6.60, N 3.54.

Synthesis of [(η^6-*p*-MeC$_6$H$_4$Pr^i)$_2$Ru$_2$(μ_2-SCH$_2$C$_6$H$_4$-*p*-Bu^t)$_2$(μ_2-SC$_6$H$_4$-*p*-CH$_2$-(C=O)-R)]Cl (R = (((1-(2-(((2*R*,3*R*,4*S*,5*R*,6R)-3,4,5-triacetoxy-6-(acetoxymethyl)tetrahydro-2*H*-pyran-2-yl)oxy)ethyl)-1*H*-1,2,3-triazol-4-yl)methyl)amino)) (24)

To a solution of **8** (0.173 g, 0.415 mmol, 1.2 equiv.) in dry DMF (10 mL) at r.t. under inert atmosphere (N$_2$) were successively added CuSO$_4$·5H$_2$O (0.086 g, 0.3444 mmol, 1 equiv.) and sodium ascorbate (0.137 g, 0.694 mmol, 2 equiv.). After 10 min, **14** (0.3698 g, 0.0346 mmol, 1 equiv.) was also added and the reaction mixture was stirred overnight at r.t.. The mixture solubilized in EtOAc (100 mL), and the organic phase was washed with water (2x100 mL), brine (100 mL), dried over Na$_2$SO$_4$,

filtered and concentrated under reduced pressure. Purification by column chromatography using a mixture CH_2Cl_2/MeOH (10:1, v/v) as eluent afforded **24** as an orange solid (0.3945 g, 0.2654 mmol, 76%).

^{1}H-NMR ($CDCl_3$) δ_H, ppm: 9.07 (1H, t br, S-(*Ar*)C-CH-CH-C-CH$_2$-(C=O)-N*H*, $^3J_{H,H}$ = 4.5 Hz), 8.31 (1H, s, (C=O)-NH-CH$_2$-(*Tr*)C-C*H*), 7.60 (2H, d, 2xS-(*Ar*)C-CH-C*H*-C-CH$_2$-(C=O), $^3J_{H,H}$ = 8.2 Hz), 7.54 (2H, d, 2xS-(*Ar*)C-C*H*-CH-C-CH$_2$-(C=O), $^3J_{H,H}$ = 7.9 Hz), 7.35-7.50 (8H, m, 4xS-CH$_2$-(*Ar*)C-C*H*-CH-C-C(CH$_3$)$_3$, 4xS-CH$_2$-(*Ar*)C-CH-C*H*-C-C(CH$_3$)$_3$, $^3J_{H,H}$ = 8.3 Hz), 5.17 (1H, t, (*Tr*)N-(CH$_2$)$_2$-O-CH-CH-C*H*-OAc, $^3J_{H,H}$ = 9.5 Hz), 5.04 (1H, t, (*Tr*)N-(CH$_2$)$_2$-O-CH-CH-CH-C*H*-OAc, $^3J_{H,H}$ = 9.7 Hz), 5.00 (2H, d, 2xCH$_3$-(*Ar*)C-C*H*-CH-C, $^3J_{H,H}$ = 5.8 Hz), 4.95 (1H, t, (*Tr*)N-(CH$_2$)$_2$-O-CH-C*H*-OAc, $^3J_{H,H}$ = 8.2 Hz), 4.88 (2H, d, 2xCH$_3$-(*Ar*)C-CH-C*H*-C, $^3J_{H,H}$ = 5.8 Hz), 4.77 (2H, d, 2xCH$_3$-(*Ar*)C-CH-C*H*-C, $^3J_{H,H}$ = 5.8 Hz), 4.61 (1H, d, (*Tr*)N-(CH$_2$)$_2$-O-C*H*-CH-OAc, $^3J_{H,H}$ = 8.0 Hz), 4.54-4.59 (4H, m, NH-C*H$_2$*-(*Tr*)C-CH-N, 2xCH$_3$-(*Ar*)C-C*H*-CH-C), 4.51 (2H, t, (*Tr*)N-C*H$_2$*-CH$_2$-O, $^3J_{H,H}$ = 5.6 Hz), 4.26 (1H, dd, CH-C*H$_2$*-OAc, $^2J_{H,H}$ = 12.3 Hz, $^3J_{H,H}$ = 4.3 Hz), 4.19-4.26 (1H, m, (*Tr*)N-CH$_2$-C*H$_2$*-O, $^3J_{H,H}$ = 5.2 Hz), 4.11 (1H, dd, CH-C*H$_2$*-OAc, $^2J_{H,H}$ = 12.5 Hz, $^3J_{H,H}$ = 1.7 Hz), 4.05 (1H, dt, (*Tr*)N-CH$_2$-C*H$_2$*-O, $^3J_{H,H}$ = 10.5 Hz, $^3J_{H,H}$ = 6.6 Hz), 3.83 (2H, s, S-(*Ar*)C-CH-CH-C-C*H$_2$*-(C=O)-NH), 3.77 (1H, ddd, C*H*-CH$_2$-OAc, $^3J_{H,H}$ = 10.0 Hz, $^3J_{H,H}$ = 4.7 Hz, $^3J_{H,H}$ = 2.3 Hz), 3.56 (2H, s, S-C*H$_2$*-(*Ar*)C-CH-CH-C-C(CH$_3$)$_3$), 3.35 (2H, s, S-C*H$_2$*-(*Ar*)C-CH-CH-C-C(CH$_3$)$_3$), 2.08 (3H, s, O-(C=O)-C*H$_3$*), 1.99 (3H, s, O-(C=O)-C*H$_3$*), 1.97 (3H, s, O-(C=O)-C*H$_3$*), 1.95 (3H, s, O-(C=O)-C*H$_3$*), 1.90 (2H, sept, 2x(*Ar*)C-CH-CH-C-C*H*(CH$_3$)$_2$, $^3J_{H,H}$ = 6.8 Hz), 1.68 (6H, s, 2xC*H$_3$*-(*Ar*)C-CH-CH-C), 1.35 (9H, s, S-CH$_2$-(*Ar*)C-CH-CH-C-C(C*H$_3$*)$_3$), 1.33 (9H, s, S-CH$_2$-(*Ar*)C-CH-CH-C-C(C*H$_3$*)$_3$), 0.93 (6H, d, (*Ar*)C-CH-CH-C-CH(C*H$_3$*)$_2$, $^3J_{H,H}$ = 6.8 Hz), 0.88 (6H, d, (*Ar*)C-CH-CH-C-CH(C*H$_3$*)$_2$, $^3J_{H,H}$ = 6.8 Hz).

^{13}C-NMR ($CDCl_3$) δ_C, ppm: 171.4 (1C, S-(*Ar*)C-CH$_2$-(C=O)-NH), 170.9 (1C, O-(C=O)-CH$_3$), 170.2 (1C, O-(C=O)-CH$_3$), 169.7 (1C, O-(C=O)-CH$_3$), 169.6 (1C, O-(C=O)-CH$_3$), 152.01, 151.98 (2C, 2xS-CH$_2$-(*Ar*)C-CH-CH-C-C(CH$_3$)$_3$), 145.7 (1C, (C=O)-NH-CH$_2$-(*Tr*)C-CH-N), 138.4 (1C, S-(*Ar*)C-CH-CH-C-CH$_2$-(C=O)-NH), 136.7, 136.5 (2C, 2xS-CH$_2$-(*Ar*)C-CH-CH-C-C(CH$_3$)$_3$), 134.8 (1C, S-(*Ar*)C-CH-CH-C-CH$_2$-(C=O)-NH), 132.2 (2C, 2xS-(*Ar*)C-CH-CH-C-CH$_2$-(C=O)-NH), 130.7 (2C, 2xS-(*Ar*)C-CH-CH-C-CH$_2$-(C=O)-NH), 129.3, 129.1 (4C, 4xS-CH$_2$-(*Ar*)C-CH-CH-C), 125.7, 125.6 (4C, 4xS-CH$_2$-(*Ar*)C-CH-CH-C), 123.8 (1C, O-(CH$_2$)$_2$-(*Tr*)N-CH-C), 107.5 (2C, 2xCH$_3$-(*Ar*)C-CH-CH-C), 100.8 (1C, (*Tr*)N-(CH$_2$)$_2$-O-CH-CH-OAc), 100.5 (2C, 2xCH$_3$-(*Ar*)C-CH-CH-C), 83.8 (4C, 2xCH$_3$-(*Ar*)C-CH-CH-C, 2xCH$_3$-(*Ar*)C-CH-CH-C), 83.6 (2C, 2xCH$_3$-(*Ar*)C-CH-CH-C), 82.4 (2C, 2xCH$_3$-(*Ar*)C-CH-CH-C), 72.9 (1C, (*Tr*)N-(CH$_2$)$_2$-O-CH-CH-CH-OAc), 71.9 (1C, CH-CH$_2$-OAc), 71.0 (1C, (*Tr*)N-(CH$_2$)$_2$-O-CH-CH-OAc), 68.5 (1C, (*Tr*)N-(CH$_2$)$_2$-O-CH-CH-CH-CH-OAc), 67.9 (1C, (*Tr*)N-CH$_2$-CH$_2$-O), 61.9 (1C, CH-CH$_2$-OAc), 49.7 (1C, (*Tr*)N-CH$_2$-CH$_2$-O), 42.8 (1C, S-(*Ar*)C-CH-CH-C-CH$_2$-(C=O)-NH), 39.9 (1C, S-CH$_2$-(*Ar*)C-CH-CH-C-C(CH$_3$)$_3$), 39.3 (1C, S-CH$_2$-(*Ar*)C-CH-CH-C-C(CH$_3$)$_3$), 36.0 (1C, NH-CH$_2$-(*Tr*)C-CH-N), 34.91 (1C, S-CH$_2$-(*Ar*)C-CH-CH-C-C(CH$_3$)$_3$), 34.88 (1C, S-CH$_2$-(*Ar*)C-CH-CH-C-C(CH$_3$)$_3$), 31.52 (3C, S-CH$_2$-(*Ar*)C-CH-CH-C-C(CH$_3$)$_3$), 31.50 (3C, S-CH$_2$-(*Ar*)C-CH-CH-C-C(CH$_3$)$_3$), 31.0 (2C, 2x(*Ar*)CH-CH-C-CH(CH$_3$)$_2$), 23.1 (2C, (*Ar*)CH-CH-C-CH(CH$_3$)$_2$), 22.8 (2C, (*Ar*)CH-CH-C-CH(CH$_3$)$_2$),

20.91 (1C, O-(C=O)-$\underline{C}H_3$), 20.85 (1C, O-(C=O)-$\underline{C}H_3$), 20.7 (2C, 2xO-(C=O)-$\underline{C}H_3$), 18.1 (2C, 2x$\underline{C}H_3$-(*Ar*)C-CH-CH-C).

ESI-MS(+): *m/z* found 1451.3876 [M-Cl]$^+$, calcd. for $C_{69}H_{91}N_4O_{11}Ru_2S_3^+$ 1451.3928.

Elemental analysis (%): calcd. for $C_{69}H_{91}ClN_4O_{11}Ru_2S_3$ C 55.76, H 6.17, N 3.77; found C 54.90, H 6.27, N 3.51.

Synthesis of [(η^6-*p*-$MeC_6H_4Pr^i$)$_2$$Ru_2$($\mu_2$-$SCH_2C_6H_4$-*p*-$Bu^t$)$_2$($\mu_2$-$SC_6H_4$-*o*-$CH_2$-R)]Cl (R = 2-(4-(3-(((2*R*,3*R*,4S,5*R*,6*R*)-3,4,5-triacetoxy-6-(acetoxymethyl)tetrahydro-2*H*-pyran-2-yl)oxy)propyl)-1*H*-1,2,3-triazol-1-yl)) (25)

To a solution of **9** (0.250 g, 0.242 mmol, 1 equiv.) in dry DMF (10 mL) at r.t. under inert atmosphere (N_2) were successively added **17** (0.200 g, 0.484 mmol, 2 equiv.), $CuSO_4·5H_2O$ (0.060 g, 0.242 mmol, 1 equiv.) and sodium ascorbate (0.096 g, 0.484 mmol, 2 equiv.). The reaction mixture was further stirred at r.t. for 24 h, then it was diluted with EtOAc (100 mL), washed with H_2O (2x100 mL), and the aqueous phase was further extracted with EtOAc (2x100 mL). The organic phases were combined, washed with brine (100 mL), dried over anhydrous Na_2SO_4, filtered, and concentrated under reduced pressure. Purification by column chromatography using CH_2Cl_2/CH_3OH 10:1 (v/v) afforded **25** as an orange solid (0.179 g, 0.125 mmol, yield 51%).

^{1}H-NMR ($CDCl_3$) δ_H, ppm: 8.63 (1H, s br, O-$(CH_2)_3$-(*Tr*)C-C$\underline{H}$-N), 7.74 (1H, dd, S-(*Ar*)C-C$\underline{H}$-CH-CH-CH-C-CH_2-N(*Tr*), $^3J_{H,H}$ = 7.9 Hz, $^4J_{H,H}$ = 1.1 Hz), 7.57 (2H, d, 2xS-CH_2-(*Ar*)C-CH-C$\underline{H}$-C-C$(CH_3)_3$, $^3J_{H,H}$ = 8.1 Hz), 7.48 (2H, d, 2xS-CH_2-(*Ar*)C-C$\underline{H}$-CH-C-C$(CH_3)_3$, $^3J_{H,H}$ = 8.2 Hz), 7.39-7.46 (4H, m, 2xS-CH_2-(*Ar*)C-C$\underline{H}$-CH-C-C$(CH_3)_3$, 2xS-CH_2-(*Ar*)C-CH-C$\underline{H}$-C-C$(CH_3)_3$, $^3J_{H,H}$ = 8.7 Hz), 7.29 (1H, td, S-(*Ar*)C-CH-CH-C$\underline{H}$-CH-C-CH_2-N(*Tr*)), $^3J_{H,H}$ = 7.4 Hz, $^4J_{H,H}$ = 0.8 Hz), 7.18-7.25 (2H, m, S-(*Ar*)C-CH-CH-CH-C$\underline{H}$-C-CH_2-N(*Tr*), S-(*Ar*)C-CH-C$\underline{H}$-CH-CH-C-CH_2-N(*Tr*), $^3J_{H,H}$ = 7.5 Hz, $^4J_{H,H}$ = 1.4 Hz), 6.45 (1H, d, S-(*Ar*)C-C-C$\underline{H}_2$-N(*Tr*), $^2J_{H,H}$ = 15.1 Hz), 6.39 (1H, d, S-(*Ar*)C-C-C$\underline{H}_2$-N(*Tr*), $^2J_{H,H}$ = 15.4 Hz), 5.20 (1H, t, (*Tr*)C-$(CH_2)_3$-O-CH-CH-C$\underline{H}$-OAc, $^3J_{H,H}$ = 9.5 Hz), 5.03-5.15 (5H, m, 2xCH_3-(*Ar*)C-C$\underline{H}$-CH-C, 2xCH_3-(*Ar*)C-CH-C$\underline{H}$-C, (*Tr*)C-$(CH_2)_3$-O-CH-CH-CH-C$\underline{H}$-OAc), 4.97 (1H, dd, (*Tr*)C-$(CH_2)_3$-O-CH-C$\underline{H}$-OAc, $^3J_{H,H}$ = 8.0 Hz, $^3J_{H,H}$ = 9.5 Hz), 4.94 (1H, d, CH_3-(*Ar*)C-CH-C$\underline{H}$-C, $^3J_{H,H}$ = 6.2 Hz), 4.91 (1H, d, CH_3-(*Ar*)C-CH-C$\underline{H}$-C, $^3J_{H,H}$ = 5.8 Hz), 4.64 (1H, d, CH_3-(*Ar*)C-C$\underline{H}$-CH-C, $^3J_{H,H}$ = 6.2 Hz), 4.62 (1H, d, CH_3-(*Ar*)C-C$\underline{H}$-CH-C, $^3J_{H,H}$ = 6.1 Hz), 4.57 (1H, d, (*Tr*)C-$(CH_2)_3$-O-C$\underline{H}$-CH-OAc, $^3J_{H,H}$ = 8.0 Hz), 4.26 (1H, dd, CH-C$\underline{H}_2$-OAc $^2J_{H,H}$ = 12.3 Hz, $^3J_{H,H}$ = 4.7 Hz), 4.10 (1H, dd, CH-C$\underline{H}_2$-OAc, $^2J_{H,H}$ = 12.3 Hz, $^3J_{H,H}$ = 2.1 Hz), 3.94 (1H, dt, O-C$\underline{H}_2$-$(CH_2)_2$-(*Tr*)C, $^2J_{H,H}$ = 9.8 Hz , $^3J_{H,H}$ = 6.2 Hz), 3.73 (1H, ddd, C$\underline{H}$-CH_2-OAc, $^3J_{H,H}$ = 10.0 Hz, $^3J_{H,H}$ = 4.5 Hz, $^3J_{H,H}$ = 2.2 Hz), 3.64 (2H, s, S-C$\underline{H}_2$-(*Ar*)C-CH-CH-C-$(CH_3)_3$), 3.57-3.68 (1H, m, O-C$\underline{H}_2$-$(CH_2)_2$-(*Tr*)C, $^3J_{H,H}$ = 6.7 Hz), 3.37 (2H, s, S-C$\underline{H}_2$-(*Ar*)C-CH-CH-C-$(CH_3)_3$), 2.82 (2H, t, O-$(CH_2)_2$-C$\underline{H}_2$-(*Tr*)C, $^3J_{H,H}$ = 7.4 Hz), 1.99-2.03 (2H, m, O-CH_2-C$\underline{H}_2$-CH_2-(*Tr*)C), 2.06 (3H, s, O-(C=O)-C$\underline{H}_3$), 2.02 (3H, s, O-(C=O)-C$\underline{H}_3$), 2.00 (3H, s, O-(C=O)-C$\underline{H}_3$), 1.97 (3H, s, O-(C=O)-C$\underline{H}_3$), 1.89 (2H, sept, 2x(*Ar*)C-CH-CH-C-C$\underline{H}$$(CH_3)_2$, $^3J_{H,H}$ = 6.9 Hz), 1.63 (3H, s, C$\underline{H}_3$-(*Ar*)C-CH-CH-C), 1.62 (3H, s, C$\underline{H}_3$-(*Ar*)C-CH-CH-C), 1.35 (9H, s, S-CH_2-(*Ar*)C-CH-CH-C-C(C$\underline{H}_3$)$_3$), 1.34 (9H, s, S-CH_2-(*Ar*)C-CH-CH-C-C(C$\underline{H}_3$)$_3$),

0.93 (6H, d, (*Ar*)C-CH-CH-C-CH(C$\underline{H}_3$)$_2$, $^3J_{H,H}$ = 6.8 Hz), 0.89 (6H, d, (*Ar*)C-CH-CH-C-CH(C$\underline{H}_3$)$_2$, $^3J_{H,H}$ = 6.9 Hz).

^{13}C-NMR (CDCl$_3$) δ_C, ppm: 170.9 (1C, O-($\underline{C}$=O)-CH$_3$), 170.3 (1C, O-($\underline{C}$=O)-CH$_3$), 169.6 (2C, 2xO-($\underline{C}$=O)-CH$_3$), 151.8, 151.6 (2C, 2xS-CH$_2$-(*Ar*)C-CH-CH-$\underline{C}$-C(CH$_3$)$_3$), 146.6 (1C, O-(CH$_2$)$_3$-(*Tr*)$\underline{C}$-CH-N), 139.9 (1C, S-(*Ar*)C-$\underline{C}$-CH$_2$-(*Tr*)N), 137.9 (1C, S-(*Ar*)$\underline{C}$-C-CH$_2$-(*Tr*)N), 136.9, 136.6 (2C, 2xS-CH$_2$-(*Ar*)$\underline{C}$-CH-CH-C-C(CH$_3$)$_3$), 133.8 (1C, S-(*Ar*)C-$\underline{C}$H-CH-CH-CH-C-CH$_2$-(*Tr*)N), 130.2 (1C, S-(*Ar*)C-CH-$\underline{C}$H-CH-CH-C-CH$_2$-(*Tr*)N), 129.9 (2C, 2xS-CH$_2$-(*Ar*)C-$\underline{C}$H-CH-C-C(CH$_3$)$_3$), 129.1 (3C, 2xS-CH$_2$-(*Ar*)C-$\underline{C}$H-CH-C-C(CH$_3$)$_3$, S-(*Ar*)C-CH-CH-$\underline{C}$H-CH-C-CH$_2$-(*Tr*)N), 128.7 (1C, S-(*Ar*)C-CH-CH-CH-$\underline{C}$H-C-CH$_2$-(*Tr*)N), 125.7, 125.5 (4C, 4xS-CH$_2$-(*Ar*)C-CH-$\underline{C}$H-C-C(CH$_3$)$_3$), 124.6 (1C, (1C, S-(*Ar*)C-C-CH$_2$-(*Tr*)N-$\underline{C}$H-C)), 106.8 (2C, 2xCH$_3$-(*Ar*)C-CH-CH-$\underline{C}$), 101.2 (1C, (*Tr*)C-(CH$_2$)$_3$-O-$\underline{C}$H-CH-OAc), 100.9 (2C, 2xCH$_3$-(*Ar*)$\underline{C}$-CH-CH-C), 83.7 (2C, 2xCH$_3$-(*Ar*)C-CH-C$\underline{H}$-C), 83.6 (4C, 2xCH$_3$-(*Ar*)C-$\underline{C}$H-CH-C, 2xCH$_3$-(*Ar*)C-CH-C$\underline{H}$-C), 82.7 (2C, 2xCH$_3$-(*Ar*)C-$\underline{C}$H-CH-C), 73.1 (1C, (*Tr*)C-(CH$_2$)$_3$-O-CH-CH-$\underline{C}$H-OAc), 71.8 (1C, $\underline{C}$H-CH$_2$-OAc), 71.5 (1C, (*Tr*)C-(CH$_2$)$_3$-O-CH-$\underline{C}$H-OAc), 69.5 (1C, (*Tr*)N-(CH$_2$)$_2$-$\underline{C}$H$_2$-O), 68.6 (1C, (*Tr*)C-(CH$_2$)$_3$-O-CH-CH-CH-$\underline{C}$H-OAc), 62.1 (1C, CH-$\underline{C}$H$_2$-OAc), 52.8 (1C, S-(*Ar*)C-C-$\underline{C}$H$_2$-N(*Tr*)), 40.4 (1C, S-$\underline{C}$H$_2$-(*Ar*)C-CH-CH-C-C(CH$_3$)$_3$), 39.1 (1C, S-$\underline{C}$H$_2$-(*Ar*)C-CH-CH-C-C(CH$_3$)$_3$), 34.9 (2C, 2xS-CH$_2$-(*Ar*)C-CH-CH-C-$\underline{C}$(CH$_3$)$_3$), 31.6, 31.5 (6C, S-CH$_2$-(*Ar*)C-CH-CH-C-C($\underline{C}$H$_3$)$_3$), 30.9 (2C, 2xCH$_3$-(*Ar*)C-CH-CH-C-$\underline{C}$H(CH$_3$)$_2$), 29.4 (1C, (*Tr*)C-CH$_2$-$\underline{C}$H$_2$-CH$_2$-O), 23.1 (2C, (*Ar*)CH-CH-C-CH-($\underline{C}$H$_3$)$_2$), 22.7 (2C, (*Ar*)CH-CH-C-CH-($\underline{C}$H$_3$)$_2$), 22.2 (1C, (*Tr*)C-$\underline{C}$H$_2$-(CH$_2$)$_2$-O), 20.90 (1C, O-(C=O)-$\underline{C}$H$_3$), 20.88 (1C, O-(C=O)-$\underline{C}$H$_3$), 20.7 (2C, 2xO-(C=O)-$\underline{C}$H$_3$), 18.2 (2C, 2x$\underline{C}$H$_3$-(*Ar*)C-CH-CH).

R_f(CH$_2$Cl$_2$/CH$_3$OH 10:1 (v/v)) = 0.500.

ESI-MS(+): *m/z* found 1408.3882 [M-Cl]$^+$, calcd. for $C_{68}H_{90}N_3O_{10}Ru_2S_3^+$ 1408.3870.

Elemental analysis (%): calcd. for $C_{68}H_{90}ClN_3O_{10}Ru_2S_3 \cdot 0.29CH_2Cl_2$ C 55.88, H 6.22, N 2.86; found C 55.89, H 6.43, N 2.81.

Synthesis of [(η^6-*p*-MeC$_6$H$_4$Pr^i)$_2$Ru$_2$(μ_2-SCH$_2$C$_6$H$_4$-*p*-Bu^t)$_2$(μ_2-SC$_6$H$_4$-*o*-CH$_2$-R)]Cl (R = 2-(4-(3-(((2*R*,3*R*,4*S*,5*S*,6*R*)-3,4,5-triacetoxy-6-(acetoxymethyl)tetrahydro-2*H*-pyran-2-yl)oxy)propyl)-1*H*-1,2,3-triazol-1-yl)) (26)

To a solution of **9** (0.250 g, 0.242 mmol, 1 equiv.) in dry DMF (10 mL) at r.t. under inert atmosphere (N$_2$) were successively added **18** (0.200 g, 0.484 mmol, 2 equiv.), $CuSO_4 \cdot 5H_2O$ (0.060 g, 0.242 mmol, 1 equiv.) and sodium ascorbate (0.096 g, 0.484 mmol, 2 equiv.). The reaction mixture was further stirred at r.t. for 24 h, then it was diluted with EtOAc (100 mL), washed with H_2O (2x100 mL), and the aqueous phase was further extracted with EtOAc (2x100 mL). The organic phases were combined, washed with brine (100 mL), dried over anhydrous Na_2SO_4, filtered, and concentrated under reduced pressure. Purification by column chromatography using CH$_2$Cl$_2$/CH$_3$OH mixture as eluent afforded **26** as an orange solid (0.223 g, 0.152 mmol, yield 63%).

^{1}H-NMR (CDCl$_3$) δ_H, ppm: 8.66 (1H, s br, S-(*Ar*)C-C-CH$_2$-(*Tr*)N-C$\underline{H}$-C), 7.74 (1H, dd, S-(*Ar*)C-C$\underline{H}$-CH-CH-CH-C-CH$_2$-N(*Tr*), $^3J_{H,H}$ = 7.8 Hz, $^4J_{H,H}$ = 1.1 Hz), 7.57 (2H, d, 2xS-CH$_2$-(*Ar*)C-CH-C$\underline{H}$-C-C(CH$_3$)$_3$, $^3J_{H,H}$ = 8.2 Hz), 7.48 (2H, d, 2xS-CH$_2$-(*Ar*)C-C$\underline{H}$-CH-C-C(CH$_3$)$_3$, $^3J_{H,H}$ = 8.3 Hz), 7.39-7.45 (4H,

m, 2xS-CH$_2$-(*Ar*)C-C*H*-CH-C-C(CH$_3$)$_3$, 2xS-CH$_2$-(*Ar*)C-CH-C*H*-C-C(CH$_3$)$_3$, $^3J_{H,H}$ = 8.7 Hz), 7.29 (1H, td, S-(*Ar*)C-CH-CH-C*H*-CH-C-CH$_2$-N(*Tr*)), $^3J_{H,H}$ = 7.6 Hz, $^4J_{H,H}$ = 1.2 Hz), 7.22 (1H, dd, S-(*Ar*)C-CH-CH-CH-C*H*-C-CH$_2$-N(*Tr*), $^3J_{H,H}$ = 7.6 Hz, $^4J_{H,H}$ = 1.5 Hz), 7.20 (1H, td, S-(*Ar*)C-CH-C*H*-CH-CH-C-CH$_2$-N(*Tr*)), $^3J_{H,H}$ = 7.3 Hz, $^4J_{H,H}$ = 1.0 Hz), 6.47 (1H, d, S-(*Ar*)C-CH-CH-CH-CH-C-C*H*$_2$-N(*Tr*), $^2J_{H,H}$ = 15.3 Hz), 6.41 (1H, d, S-(*Ar*)C-CH-CH-CH-CH-C-C*H*$_2$-N(*Tr*), $^2J_{H,H}$ = 15.6 Hz), 5.37 (1H, dd, (*Tr*)C-(CH$_2$)$_3$-O-CH-CH-CH-C*H*-OAc, $^3J_{H,H}$ = 3.3 Hz, $^3J_{H,H}$ = 0.6 Hz), 5.19 (1H, dd, (*Tr*)C-(CH$_2$)$_3$-O-CH-C*H*-OAc, $^3J_{H,H}$ = 10.5 Hz, $^3J_{H,H}$ = 7.9 Hz), 5.06-5.13 (4H, m, 2xCH$_3$-(*Ar*)C-CH-C*H*-C, 2xCH$_3$-(*Ar*)C-C*H*-CH-C), 5.02 (1H, dd, (*Tr*)C-(CH$_2$)$_3$-O-CH-CH-C*H*-OAc, $^3J_{H,H}$ = 10.5 Hz, $^3J_{H,H}$ = 3.4 Hz), 4.95 (1H, d, CH$_3$-(*Ar*)C-CH-C*H*-C, $^3J_{H,H}$ = 5.8 Hz), 4.91 (1H, d, CH$_3$-(*Ar*)C-CH-C*H*-C, $^3J_{H,H}$ = 5.8 Hz), 4.64 (1H, d, CH$_3$-(*Ar*)C-C*H*-CH-C, $^3J_{H,H}$ = 6.1 Hz), 4.62 (1H, d, CH$_3$-(*Ar*)C-C*H*-CH-C, $^3J_{H,H}$ = 6.2 Hz), 4.55 (1H, d, (*Tr*)C-(CH$_2$)$_3$-O-C*H*-CH-OAc, $^3J_{H,H}$ = 8.0 Hz), 4.13 (2H, d, CH-C*H*$_2$-OAc, $^3J_{H,H}$ = 6.7 Hz), 3.93-3.99 (1H, m, (*Tr*)C-(CH$_2$)$_2$-C*H*$_2$-O, $^3J_{H,H}$ = 6.1 Hz), 3.93 (1H, t, C*H*-CH$_2$-OAc, $^3J_{H,H}$ = 6.5 Hz), 3.64 (2H, s, S-C*H*$_2$-(*Ar*)C-CH-CH-C-(CH$_3$)$_3$), 3.57-3.68 (1H, m, (*Tr*)C-(CH$_2$)$_2$-C*H*$_2$-O, $^3J_{H,H}$ = 6.9 Hz), 3.37 (2H, s, S-C*H*$_2$-(*Ar*)C-CH-CH-C-(CH$_3$)$_3$), 2.82 (2H, t, (*Tr*)C-C*H*$_2$-(CH$_2$)$_2$-O, $^3J_{H,H}$ = 7.5 Hz), 2.12 (3H, s, O-(C=O)-C*H*$_3$), 1.98-2.06 (2H, m, (*Tr*)C-CH$_2$-C*H*$_2$-CH$_2$-O), 2.03 (3H, s, O-(C=O)-C*H*$_3$), 2.02 (3H, s, O-(C=O)-C*H*$_3$), 1.96 (3H, s, O-(C=O)-C*H*$_3$), 1.89 (2H, sept, 2x(*Ar*)C-CH-CH-C-C*H*(CH$_3$)$_2$, $^3J_{H,H}$ = 6.9 Hz), 1.63 (3H, s, C*H*$_3$-(*Ar*)C-CH-CH-C), 1.62 (3H, s, C*H*$_3$-(*Ar*)C-CH-CH-C), 1.35 (9H, s, S-CH$_2$-(*Ar*)C-CH-CH-C-C(C*H*$_3$)$_3$), 1.34 (9H, s, S-CH$_2$-(*Ar*)C-CH-CH-C-C(C*H*$_3$)$_3$), 0.93 (6H, d, (*Ar*)C-CH-CH-C-CH(C*H*$_3$)$_2$, $^3J_{H,H}$ = 6.9 Hz), 0.89 (6H, d, (*Ar*)C-CH-CH-C-CH(C*H*$_3$)$_2$, $^3J_{H,H}$ = 6.9 Hz).

^{13}C-NMR (CDCl$_3$) δ_C, ppm: 170.6 (1C, O-(C=O)-CH$_3$), 170.4 (1C, O-(C=O)-CH$_3$), 170.2 (1C, O-(C=O)-CH$_3$), 169.7 (1C, O-(C=O)-CH$_3$), 151.8, 151.6 (2C, 2xS-CH$_2$-(*Ar*)C-CH-CH-C-C(CH$_3$)$_3$), 146.6 (1C, S-(*Ar*)C-C-CH$_2$-(*Tr*)N-CH-C), 140.0 (1C, S-(*Ar*)C-C-CH$_2$-(*Tr*)N-CH-C), 137.8 (1C, S-(*Ar*)C-C-CH$_2$-(*Tr*)N-CH-C), 136.9, 136.6 (2C, 2xS-CH$_2$-(*Ar*)C-CH-CH-C-C(CH$_3$)$_3$), 133.7 (1C, S-(*Ar*)C-CH-CH-CH-CH-C-CH$_2$-N(*Tr*)), 130.2 (1C, S-(*Ar*)C-CH-CH-CH-CH-C-CH$_2$-N(*Tr*)), 129.9 (2C, 2xS-CH$_2$-(*Ar*)C-CH-CH-C-C(CH$_3$)$_3$), 129.1 (3C, 2xS-CH$_2$-(*Ar*)C-CH-CH-C-C(CH$_3$)$_3$, S-(*Ar*)C-CH-CH-CH-CH-C-CH$_2$-N(*Tr*)), 128.7 (1C, S-(*Ar*)C-CH-CH-CH-CH-C-CH$_2$-N(*Tr*)), 125.7, 125.5 (4C, 4xS-CH$_2$-(*Ar*)C-CH-CH-C-C(CH$_3$)$_3$), 124.6 (1C, S-(*Ar*)C-C-CH$_2$-(*Tr*)N-CH-C), 106.8 (2C, 2xCH$_3$-(*Ar*)C-CH-CH-C), 101.4 (2C, 2xCH$_3$-(*Ar*)C-CH-CH-C), 101.2 (1C, (*Tr*)C-(CH$_2$)$_3$-O-C*H*-CH-OAc), 83.8, 83.7 (2C, 2xCH$_3$-(*Ar*)C-CH-CH-C), 83.6, 83.5 (4C, 4xCH$_3$-(*Ar*)C-CH-C*H*-C), 82.7 (2C, 2xCH$_3$-(*Ar*)C-CH-CH-C), 71.2 (1C, (*Tr*)C-(CH$_2$)$_3$-O-CH-CH-CH-OAc), 70.7 (1C, CH-CH$_2$-OAc), 69.5 (1C, (*Tr*)C-(CH$_2$)$_2$-CH$_2$-O), 69.1 (1C, (*Tr*)C-(CH$_2$)$_3$-O-CH-CH-OAc), 67.3 (1C, (*Tr*)C-(CH$_2$)$_3$-O-CH-CH-CH-CH-OAc), 61.4 (1C, CH-CH$_2$-OAc), 52.8 (1C, S-(*Ar*)C-C-CH$_2$-(*Tr*)N), 40.4 (1C, S-CH$_2$-(*Ar*)C-CH-CH-C-C(CH$_3$)$_3$), 39.1 (1C, S-CH$_2$-(*Ar*)C-CH-CH-C-C(CH$_3$)$_3$), 34.9 (2C, 2xS-CH$_2$-(*Ar*)C-CH-CH-C-C(CH$_3$)$_3$), 31.6 (3C, S-CH$_2$-(*Ar*)C-CH-CH-C-C(CH$_3$)$_3$), 31.5 (3C, S-CH$_2$-(*Ar*)C-CH-CH-C-C(CH$_3$)$_3$), 30.9 (2C, 2xCH$_3$-(*Ar*)C-CH-CH-C-CH(CH$_3$)$_2$), 29.5 (1C, (*Tr*)C-CH$_2$-CH$_2$-CH$_2$-O), 23.1 (2C, (*Ar*)CH-CH-C-CH-(CH$_3$)$_2$), 22.8 (2C, (*Ar*)CH-CH-C-CH-(CH$_3$)$_2$), 22.2 (1C, (*Tr*)C-CH$_2$-(CH$_2$)$_2$-O), 21.0 (1C, O-(C=O)-CH$_3$), 20.83 (1C, O-(C=O)-CH$_3$), 20.81 (1C, O-(C=O)-CH$_3$), 20.7 (1C, O-(C=O)-CH$_3$), 18.2 (2C, 2xCH$_3$-(*Ar*)C-CH-CH).

R_f(CH$_2$Cl$_2$/CH$_3$OH 10:1 (v/v)) = 0.468.

ESI-MS(+): *m*/*z* found 1408.3868 [M-Cl]$^+$, calcd. for $C_{68}H_{90}N_3O_{10}Ru_2S_3^+$ 1408.3870.

Elemental analysis (%): calcd. for $C_{68}H_{90}ClN_3O_{10}Ru_2S_3 \cdot 0.1CH_2Cl_2 \cdot 2.2H_2O$ C 54.85, H 6.39, N 2.82; found C 54.86, H 6.40, N 2.79.

Stability in DMSO-*d*$_6$

A

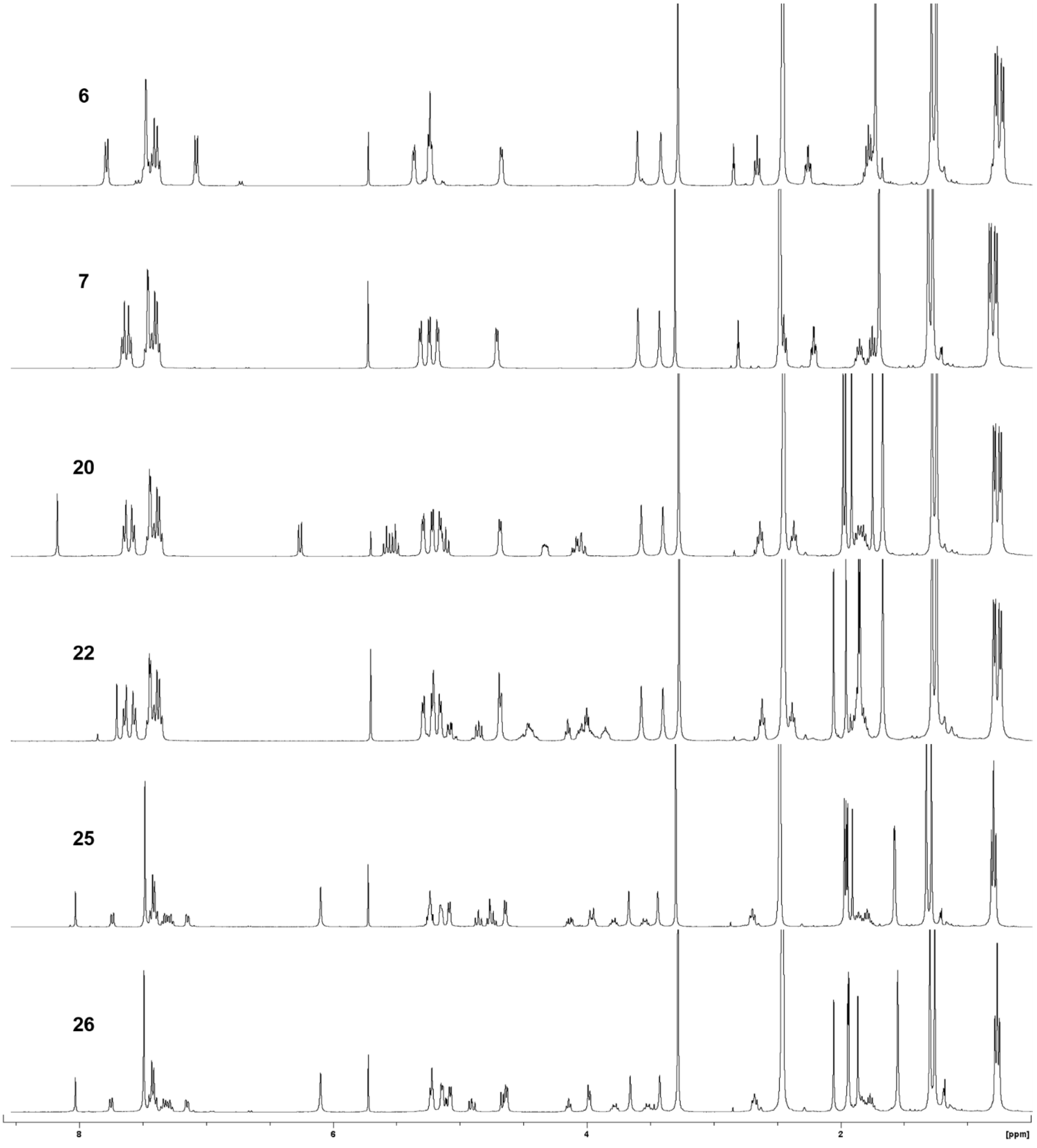

B

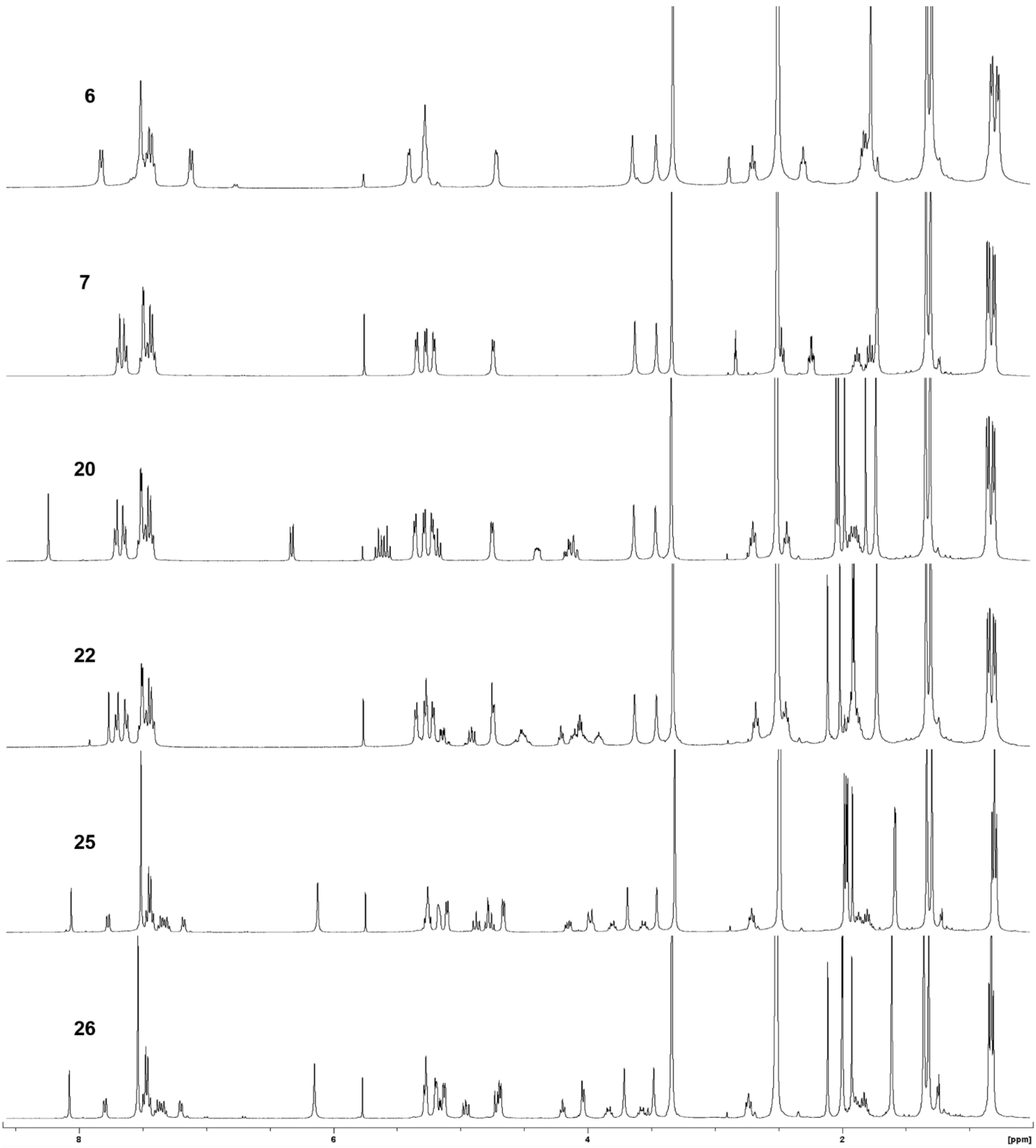

Figure S1. ^{1}H NMR Spectra of **6**, **7**, **20**, **22**, **25** and **26** recorded in DMSO-d_6 at 25°C; (A) recorded 5 min after sample preparation, and (B) sample after > 30 days storage at 0–5°C in the dark.

References

1. Fulmer, G.R.M., A.J.M.; Sherden, N.H.; Gottlieb, H.E.; Nudelman, A.; Stoltz, B.M.; Bercaw, J.E.; Goldberg, K.I. NMR chemical shifts of trace impurities: Common laboratory solvents, organics, and gases in deuterated solvents relevant to the organometallic chemist. *Organometallics* **2010**, *29*, 2176–2179. https://doi.org/10.1021/om100106e.
2. Desiatkina, O.; Paunescu, E.; Mosching, M.; Anghel, N.; Boubaker, G.; Amdouni, Y.; Hemphill, A.; Furrer, J. Coumarin-tagged dinuclear trithiolato-bridged ruthenium(II)arene complexes: Photophysical properties and antiparasitic activity. *ChemBioChem* **2020**, *21*, 2818–2835. https://doi.org/10.1002/cbic.202000174.
3. Giannini, F.; Furrer, J.; Süss-Fink, G.; Clavel, C.M.; Dyson, P.J. Synthesis, characterization and in vitro anticancer activity of highly cytotoxic trithiolato diruthenium complexes of the type [(η6-p-MeC6H4iPr)2Ru2(μ2-SR1)2(μ2-SR2)]+ containing different thiolato bridges. *J. Organomet. Chem.* **2013**, *744*, 41–48. https://doi.org/10.1016/j.jorganchem.2013.04.049.
4. Păunescu, E.B., G.; Desiatkina, O.; Anghel, N.; Amdouni, Y.; Hemphill, A.; Furrer, J. The quest of the best-A SAR study of trithiolato-bridged dinuclear ruthenium(II)-arene compounds presenting antiparasitic properties. *Eur. J. Med. Chem.* **2021**, *222*, 113610. https://doi.org/10.1016/j.ejmech.2021.113610.
5. Desiatkina, O.; Johns, S.K.; Anghel, N.; Boubaker, G.; Hemphill, A.; Furrer, J.; Păunescu, E. Synthesis and antiparasitic activity of new conjugates-organic drugs tethered to trithiolato-bridged dinuclear ruthenium(II)-arene complexes. *Inorganics* **2021**, *9*, 59. https://doi.org/10.3390/inorganics9080059.
6. Desiatkina, O.; Mösching, M.; Anghel, N.; Boubaker, G.; Amdouni, Y.; Hemphill, A.; Furrer, J.; Păunescu, E. New nucleic base-tethered trithiolato-bridged binuclear ruthenium(II)-arene compounds: Synthesis and antiparasitic activity. *Molecules* **2022**, *27*, 8173. https://doi.org/10.3390/molecules27238173.
7. Soli, E.D.; DeShong, P. Advances in glycosyl azide preparation via hypervalent silicates. *J. Org. Chem.* **1999**, *64*, 9724–9726. https://doi.org/10.1021/jo991161s.
8. Ogawa, T.; Nakabayashi, S.; Shibata, S. Synthetic studies on nephritogenic glycosides. Synthesis of N-(β-l-Aspartyl)-α-d-glucopyranosylamine. *Agric. Biol. Chem.* **1983**, *47*, 281–285. https://doi.org/10.1080/00021369.1983.10865618.
9. Patra, M.; Awuah, S.G.; Lippard, S.J. Chemical approach to positional isomers of glucose–platinum conjugates reveals specific cancer targeting through glucose-transporter-mediated uptake in vitro and in vivo. *J. Am. Chem. Soc.* **2016**, *138*, 12541–12551. https://doi.org/10.1021/jacs.6b06937.
10. Paterson, S.M.; Clark, J.; Stubbs, K.A.; Chirila, T.V.; Baker, M.V. Carbohydrate-based crosslinking agents: Potential use in hydrogels. *J. Polym. Sci., Part A: Polym. Chem.* **2011**, *49*, 4312–4315. https://doi.org/10.1002/pola.24892.
11. Mereyala, H.B.; Gurrala, S.R. A highly diastereoselective, practical synthesis of allyl, propargyl 2,3,4,6-tetra-O-acetyl-β-d-gluco, β-d-galactopyranosides and allyl, propargyl heptaacetyl-β-d-lactosides. *Carbohydrate Res.* **1998**, *307*, 351–354. https://doi.org/10.1016/S0008-6215(97)10104-5.
12. Roy, B.; Dutta, S.; Choudhary, A.; Basak, A.; Dasgupta, S. Design, synthesis and RNase A inhibition activity of catechin and epicatechin and nucleobase chimeric molecules. *Bioorg. Med. Chem. Lett.* **2008**, *18*, 5411–5414. https://doi.org/10.1016/j.bmcl.2008.09.051.
